# Molecular characterization of the archaic *HLA-B∗73:01* allele reveals presentation of a unique peptidome and skewed engagement by KIR2DL2

**DOI:** 10.1016/j.jbc.2025.110542

**Published:** 2025-07-30

**Authors:** Philipp. Ross, Hugo G. Hilton, Jane Lodwick, Tomasz Slezak, Lisbeth A. Guethlein, Curtis P. McMurtrey, Alex S. Han, Morten Nielsen, Daniel Yong, Charles L. Dulberger, Kristof T. Nolan, Sobhan Roy, Caitlin D. Castro, William H. Hildebrand, Minglei Zhao, Anthony Kossiakoff, Peter Parham, Erin J. Adams

**Affiliations:** 1 Department of Biochemistry and Molecular Biology, The University of Chicago, Chicago, Illinois, USA; 2 Committee on Genetics, Genomics and Systems Biology, The University of Chicago, Chicago, Illinois, USA; 3 Department of Structural Biology, School of Medicine, Stanford University, Stanford, USA; 4 Department of Microbiology & Immunology, School of Medicine, Stanford University, Stanford, USA; 5 Department of Microbiology & Immunology, University of Oklahoma Health Sciences Center, Oklahoma City, Oklahoma, USA; 6 Department of Health Technology, Technical University of Denmark, Kgs. Lyngby, Denmark

**Keywords:** natural killer cells, MHC, comparative immunology/evolution, antigens/peptides/epitopes

## Abstract

HLA class I alleles of archaic origin may have been retained in modern humans because they provide immunity against diseases to which archaic humans had evolved resistance. According to this model, archaic introgressed alleles were somehow distinct from those that evolved in African populations. Here, we show that HLA-B∗73:01, a rare allotype with putative archaic origins, has a relatively rare peptide binding motif with an unusually long-tailed peptide length distribution. We also find that HLA-B∗73:01 combines a restricted and unique peptidome with high-cell surface expression, characteristics that make it well-suited to combat one or a number of closely related pathogens. Furthermore, a crystal structure of HLA-B∗73:01 in complex with KIR2DL2 highlights differences from previously solved structures with HLA-C molecules. These molecular characteristics distinguish HLA-B∗73:01 from other HLA class I alleles previously investigated and may have provided early modern human migrants that inherited this allele with a selective advantage as they colonized Europe and Asia.

When modern humans migrated out of Africa they hybridized with archaic humans, including Neanderthals and Denisovans, that were already resident in Europe and Asia (1, 2, 3, 4). As a result, 1.5 to 6% of modern non-African human genomes derive from archaic humans. In general, purifying selection has acted to remove archaic human DNA from modern humans (5). However, a limited number of introgressed archaic human genes have been preferentially retained (6, 7, 8). Among these adaptive loci are several dedicated to immune function, including selected alleles of the highly polymorphic HLA class I genes (*HLA*-A, *-B*, and *-C*) (6). HLA class I molecules are present on the surface of all nucleated cells and are one of two primary classes of molecules encoded by the major histocompatibility complex (MHC), the other being HLA class II. Their function is to display peptide fragments of varying length from within the cell for outward immunosurveillance by T cells and natural killer (NK) cells, allowing the immune system to differentiate healthy from diseased tissue. The variety and type of peptides bound by an HLA class I molecule depends on the amino acid sequence of its peptide binding groove and underlies the correlation of specific HLA class I alleles with protection or susceptibility to a range of autoimmune and infectious diseases.

Admixture with archaic humans is hypothesized to have restored *HLA* diversity in modern humans following the population bottleneck that occurred during the out of Africa migration and may have been a route to acquire advantageous HLA variants already adapted to local pathogens (6, 9). A putative archaic HLA class I allele of particular interest is *HLA-B∗73:01* (B∗73:01). This exceptional allele, the only member of a deeply divergent lineage (MHC-BII), is distinct from the MHC-BI lineage to which all other human *HLA-B* alleles belong and is more closely related to subsets of chimpanzee and gorilla MHC-B than other human *HLA-B* alleles (6, 10). Further distinguishing B∗73:01 is that it is one of only two of the HLA-B classes that encodes the C1 NK cell-binding epitope formed by residues V76 and N80 in the α1 helix (11, 12). Of note, this C1 motif is only found in B∗73 and B∗46, and is found in all known allotypes of these genes (4, and 100 allotypes, respectively, out of 5745 total B proteins at last count) (13). This epitope confers reactivity with killer-cell immunoglobulin-like receptors (KIR) 2DL3 and 2DL2, inhibitory receptors that regulate the function of human NK cells. *HLA-B∗46:01* (B∗46:01), the other HLA-B allele with a C1 epitope, acquired this epitope more recently through a mini-gene conversion of *B∗15* with *HLA-C∗01:02* (14, 15) (C∗01:02). Both B∗73:01 and B∗46:01 have been shown to bind KIR2DL2 and KIR2DL3 using HLA-coupled beads and KIR-Fc fusion reagents (11, 16), and HLA-B∗46:01 expressing cells are able to functionally inhibit KIR2DL3 expressing NK cells (14).

Carriers of the C1 epitope as well as KIR2DL2 or KIR2DL3 have varying susceptibilities to certain diseases (17). One of the earliest identified examples of this was the finding that individuals that are homozygous for the C1 epitope and KIR2DL3 are better protected from chronic hepatitis C virus than individuals without C1-epitope containing HLAs or C1 carriers that also encode KIR2DL2 (18). These examples are thought to be mediated by NK cells, but KIR2DL2 is also known to be associated with adaptive immune outcomes (19). More recently, an in-depth analysis of B∗46:01 carriers in Southeast Asia showed that HIV + carriers of *B∗46:01* more rapidly progressed to AIDS than individuals without (20) and that the NK cell phenotypes of these individuals showed unusual levels of activation. These associations raise the possibility that alleles carrying the C1 epitope for KIR2DL2 or KIR2DL3 play a direct role in immunosurveillance. Although there has been extensive work in understanding the interaction of these KIRs with HLA-C alleles bearing the C1 epitope, relatively little is known about how this C1 epitope in B∗46:01 and B∗73:01 is engaged by KIRs. Furthermore, little is known about the peptide repertoire presented by B∗73:01, or how it might be associated with human disease.

Although *B∗73:01* presumably conferred some selective advantage when it was initially introduced into modern humans by adaptive introgression, the allele is now rare, indicating either that the selective pressure that drove its retention is no longer present, or that selective advantage also comes with a cost. However, due to the low frequency of *B∗73:01* in modern day human populations (6), confident genetic associations with maladies such as infectious diseases would be underpowered and therefore unreliable. Thus, to better understand why *B∗73:01* was initially retained in modern humans, we studied its peptide repertoire, cell-surface expression, and three-dimensional atomic structure alone and in complex with KIR2DL2 and compared these characteristics to other HLA alleles. Our findings indicate that *B∗73:01* encodes a protein with a highly distinctive peptide repertoire with unique peptide presentation abilities, and is engaged by KIR2DL2 with a skewed footprint, binding that is likely modulated by the length of the presented peptide. Together, these features may have important immune consequences, which underlie its adaptive introgression into modern human genomes (6, 15, 21, 22, 23).

## Results

### B∗73:01 presents a restricted peptide binding repertoire with an unusual length distribution

**Figure 1:**
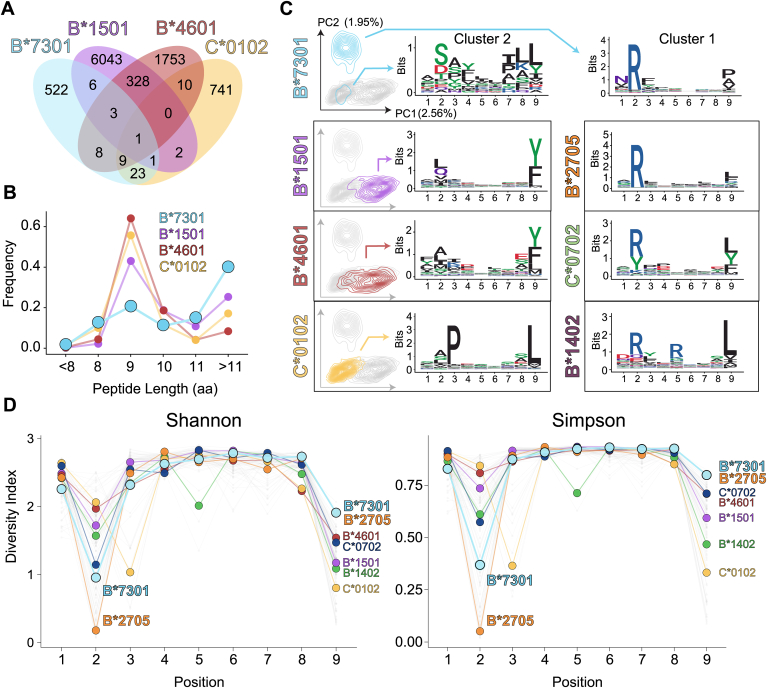
**HLA-B∗73:01 has evolved a rare peptide binding repertoire with an unusual length distribution.***A*, Venn diagram showing the number of distinct peptides eluted from the HLA class I allotypes: B∗73:01, B∗15:01, B∗46:01 and C∗01:02. The number of peptides shared with each of the other allotypes is shown. *B*, length distribution of peptides eluted from the HLA allotypes in (*A*). *C*, principal components analysis contour plots show the distribution of nonamer peptides bound by each HLA class I allotype (on *left*). For comparison, in each plot the distribution of nonamer peptides from the other allotypes is shown in *gray*. The percentage of the variation in the dataset shown by the first (PC1) and second (PC2) principal components is shown near the axes. Sequence logos of 9mers isolated from the analysis in (*A*), categorized by unsupervised alignment and clustering, are shown for peptides eluted from HLA-B∗73:01, B∗15:01, B∗46:01, and C∗01:02. Data from Sarkizova *et al.* (32) was used to generate similar plots for B∗27:05, C∗07:02, and B∗14:02 (on *right*). The height of each amino acid in the plot represents the level of conservation at that position and its relative frequency. The colors of each amino acid correspond to their biochemical characteristics: acidic (*red*), basic (*blu*e), hydrophobic (*black*), and polar (*green*). *D*, Shannon and Simpson diversity indices calculated for each position for HLA-B∗73:01, B∗15:01, B∗46:01, and C∗01:02 using peptides eluted and sequenced by mass spectrometry in our study.

To investigate the repertoire of peptides bound by B∗73:01, we purified heterogeneously loaded protein, eluted the bound peptides, and determined their sequences by LC-MS (24, 25, 26). We then compared B∗73:01 peptides with those of other well characterized alleles: *HLA-B∗15:01* (B∗15:01), the partner of the gene conversion that formed *B∗46:01*, as well as *HLA-C∗01:02* (C∗01:02), and *HLA-B∗46:01* (B∗46:01) that were all expressed, purified, and sequenced the same way in a previous study (15). Of note, since a pan class-I antibody (α-W6/32) column was used for purification, we do not anticipate that this purification would introduce biases to the eluted peptides compared to other HLA classes. With only 573 distinct peptides captured and confidently identified, B∗73:01 has the smallest of the four peptide repertoires and is an order of magnitude smaller than the >6000 peptides bound by B∗15:01 (Fig. 1*A*). Furthermore, fewer than 10% of the B∗73:01 bound peptides overlapped with one or more of the other alleles investigated. Examples of peptides eluted from B∗73:01 and used later in this analysis are listed in Table S1. Of note, while the paucity of recovered peptides from the B∗73:01 preparations might indicate a general lower affinity B∗73:01 for peptides, we believe that the smaller number of recovered peptides from B∗73:01 is more likely due to its preference for particular peptides as opposed a systematically lower affinity for all peptides. Refolding experiments performed in our lab using more than one dozen peptides (Fig. S1) show high variability and specificity depending on the peptide used. Peptides with anchors that include R at P2 and V or P at P omega, refold extremely efficiently whereas with peptides that carry a K at P2 or using a negative control peptide (FTM; reported to bind HLA-B∗46:01), refolding efficiency is severely compromised.

Contributing to the distinctiveness of B∗73:01 peptides is a considerable promiscuity in the eluted peptide length. When compared to other alleles, we found that nonamer peptides comprise only ∼20% of B∗73:01 peptides (Fig. 1*B*). Instead, the majority (∼40%) of peptides that B∗73:01 presents are greater than 11 amino acids in length, reminiscent of the length of peptides that are bound to HLA class II molecules, which, by virtue of their open-ended peptide binding groove, routinely bind longer peptides (27). In addition, on average, the length of peptides presented by B∗73:01 is significantly larger than those presented by other alleles, with average lengths of 12.36, 9.76, 9.66, and 9.38 for B∗73:01, B∗15:01, B∗46:01, and C∗01:02, respectively (Fig. S2). To understand how B∗73:01 can accommodate such long peptides, we compared the peptide binding motifs of peptides of varying lengths and found that even as the peptides get longer, they retain a canonical B∗73:01 binding motif with a preferred arginine at Position 2 (P2) and a hydrophobic residue at the peptide C terminus (Fig. S3*A*). This suggests that these positions remain the primary peptide anchors in B∗73:01 with the intervening residues accommodated by “bulging out” from the peptide binding pocket without greatly destabilizing the protein complex. Peptides of lengths above 11 amino acids were analyzed for strong HLA-B∗73:01 binding using NetMHCpan-4.1 (28). Several of these longer peptides (∼12–15 amino acids long) were predicted to exhibit high binding values (Table S1). Because nonamers represent the most common length of peptide from classical HLA I class I proteins and the nonamer sequences from B∗73:01 maintain the consensus motifs represented in the other lengths of B∗73:01 peptides, we chose to compare this length with those of other nonamers from other HLA alleles in the analysis presented below.

Visualization of the relative similarity of the nonamer peptidomes of B∗73:01, B∗15:01, B∗46:01, and C∗01:02 by principal components analysis supports the uniqueness of the B∗73:01 peptide repertoire motifs, showing that most B∗73:01 bound peptides have little spatial overlap with those of the other three alleles (Fig. 1*C* left column). The greatest contributor to PC2 is the hydropathy index (55%), a measure of the hydrophobicity/hydrophilicity of side chains; in B∗73:01 this is reflected in the strong preference for an arginine at P2 (Fig. 1*C*). Using an unsupervised alignment and clustering of peptide sequences (29, 30), we can identify two divergent peptide binding motifs for B∗73:01, with 85% of nonamers contributing to cluster 1 and the remaining peptides to cluster 2. The cluster 1 peptides maintain this P2-Arginine/P9-hydrophobic preference, while peptides from cluster 2 show less specific anchor preferences and are more like peptides bound by B∗15:01, B∗46:01, and C∗01:02. The motif represented in the Cluster 1 nonamer peptides from B∗73:01 shows strong resemblance to peptides bound by the *HLA-B∗27* family of alleles (for example B∗27:05, shown in orange in Fig. 1*C*, right panel). Of note, there is evidence that other HLA alleles with compact peptidomes, like B∗27:05, although sometimes protective, may also predispose individuals to autoimmune disease, as is the case with *B∗27:05* and axial spondyloarthritis (31).

To understand how B∗73:01 compares to HLA alleles representative of those with varying frequencies worldwide, we compared our B∗73:01 peptidome data to that from Sarkizova *et al.*, which includes >185,000 total peptides from 95 alleles, chosen such that 95% of individuals worldwide have at least one of the HLA-A, HLA-B, and HLA-C alleles included (32). The peptidome for each allele was generated using the same parental cell line (BLCL 721.221) as we used for B∗73:01, B∗15:01, B∗46:01, and C∗01:02. To confirm that we can confidently combine these two datasets together we compared the peptide binding motifs of eluted nonamers for alleles in both datasets. By doing this, we found that eluted nonamers from both studies were highly concordant with Pearson correlation values of 0.93, 0.93, and 0.99 for B∗15:01, B∗46:01, and C∗01:02, respectively (Fig. S3*B*), suggesting that the peptidome of B∗73:01 sequenced using our approach is comparable to those of Sarkizova. Of note, although our peptide repertoire analyses were performed on secreted B∗73:01, and Sarkizova *et al.*'s was from total cell lysates, similar comparisons have previously found that the peptidomes from these two sources are highly congruent (33). However, as an added check, we compared the peptidomes for alleles HLA-B∗15:01, HLA-B∗46:01, and HLA-C∗01:02, using both our method and the method described in Sarkizova *et al.*, and found that the published results from whole lysates correlate positively (Pearson) with data generated in our previous study (Fig. S3*B*). Incorporating these additional peptide repertoires into our analysis, the similarity between the peptide preferences of B∗73:01 with those of other HLA alleles became apparent. For nonamer peptides, the HLA alleles B∗27:05, C∗07:02, and B∗14:02 share commonalities in their preference for an arginine at position 2 and a hydrophobic residue (leucine) at position 9 (Fig. 1*C*, right panel).

Using a similar approach to Sarkizova *et al.* we compared B∗73:01 to this more extensive peptide repertoire analysis using the data from both peptides bound (“peptide space”) and the variable pocket residues within the platform regions of HLA molecules (“pocket space”). These analyses revealed at a more granular level which peptidomes shared the greatest similarity to that of B∗73:01. Consistent with our previous findings in Figure 1, *HLA B∗27:05* (B∗27:05) had the highest similarity to B∗73:01 in both peptide space and pocket space (Fig. S4*A*), whereas *HLA-C∗07:02* (C∗07:02), *HLA-C∗07:01* (C∗07:01) and *HLA-C∗06:02* (C∗06:02) had high similarity in peptide space (Fig. S4*A*; left panel), and *HLA-B∗56:01* (B∗56:01) and *HLA-B∗14:02* (B∗14:02) had highest similarity in terms of pocket space. B∗07:02, B∗07:04, B∗08:01, B∗13:02, B∗40:02, B∗40:06, B∗42:01, B∗54:01, B∗55:01, B∗55:02, and B∗56:01 also shared in similarity to B∗73:01 in terms of pocket space (labels not shown in figure due to space constraints) (Fig. S4*A*; right panel). Thus, similar to comparisons made with only B∗15:01, B∗46:01, and C∗01:02, the divergent nature of the B∗73:01 peptidome stems from its strong preference for arginine at P2. Reanalysis of the peptide repertoire length of B∗73:01 with this expanded dataset recapitulated our original results, notably a preference for peptides longer than 11 amino acids is a unique feature of B∗73:01 (Fig. S4*B*).

Recent reports suggest that HLAs can be categorized as either “generalists” or “specialists” depending on the promiscuity of the peptides they can present (34). To compare the diversity of these B∗73:01 characterized peptides, we calculated diversity indices using either Shannon–to highlight subtle differences in rare residues (35)–or Simpson–to assess the uniformity among the more common residues–measures at each position of the HLA alleles presented above and those from Sarkizova *et al.* (Fig. 1*D*). Interestingly, these analyses show that B∗73:01 is not a statistical outlier relative to other HLA alleles in the number of residues anchored at positions 2 or 9 (Fig. S5). In fact B∗73:01 has relatively low sequence diversity at P2 (with B∗27:05 having the lowest diversity), and only B∗27:05 and C∗12:03 have higher diversity indices at P9. Together, these data show that B∗73:01 has evolved the ability to present peptides with a restricted binding motif relative to other more common alleles, and instead, more similar to the B∗27 family of alleles. In addition, B∗73:01 peptides are proportionally longer and vary most at their C terminal anchor, while being heavily biased toward arginine at the P2 anchor position.

### B∗73:01 combines a size-restricted and unique peptidome with above average cell-surface expression

**Figure 2:**
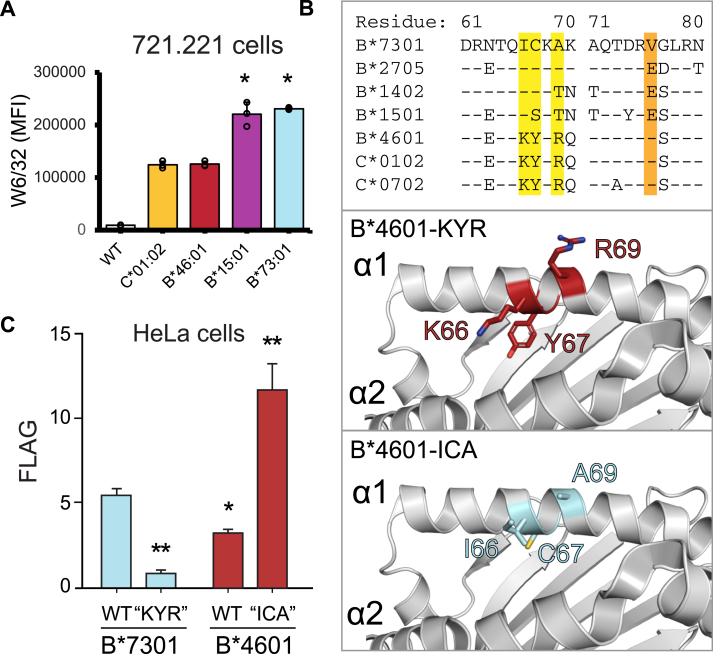
**HLA-B∗73:01 combines a size-restricted and unique peptidome with above-average cell-surface expression.***A*, MFI of cell-surface expression of four HLA class I allotypes as determined by W6/32 staining in transfected 721.221 cells (*asterisks* indicate expression that differs significantly from HLA-B∗46:01 (two-tailed *t* test, ∗ indicates *p* < 0.005)). *Dots* represent three individual technical replicates. *B*, *Toppanel*: An alignment of selected HLA amino acid sequences from residue 61 to 80, containing the KYRV motif (highlighted in *yellow* and *orange*) known to affect the cell surface expression of HLA-C alleles (38, 39). A crystal structure of HLA-B∗46:01 (PDB ID: 4LCY) showing the WT KYR motif in *red* (*middlepanel*) or the ICA motif in cyan (*lowerpanel*), following *in silico* mutagenesis in PyMol. *C*, surface expression of FLAG-tagged wild-type (WT) HLA-B∗73:01 and HLA-B∗46:01 and position 66, 67, and 69 (KYR/ICA) swap mutants in HeLa cells as determined by anti-FLAG antibody staining; *asterisks* indicate expression that differs significantly from HLA-B∗73:01-WT (two-tailed *t* test, ∗ indicates *p* < 0.05, ∗∗ indicates *p* < 0.005). Data are plotted as fold change relative to the KYR reference. MFI, median fluorescence intensity; PDB, Protein Data Bank.

Recent work suggests that peptide binding promiscuity of a given HLA class I peptidome is inversely correlated with its cell surface expression (36). Considering the restricted repertoire of the peptides presented by B∗73:01, we hypothesized that it would have concordantly high cell-surface expression levels relative to other alleles. To test this, we first investigated the expression of each of the alleles used in our original analysis (Fig. 1, *A*–*C*) (15) in 721.221 cells, which lack endogenous expression of HLA class I, using W6/32 (37), an antibody that recognizes all HLA class I alleles with equal avidity. Indeed, we find that B∗73:01 is the highest expressed allele of the four, being expressed at a significantly higher level than both C∗01:02 and B∗46:01, but at an approximately equal level to B∗15:01 (Fig. 2*A*). The low cell-surface expression of C∗01:02 is consistent with previous studies showing that alleles of the HLA-C locus are expressed at a significantly lower level at the cell-surface than alleles of the HLA-A or HLA-B loci (38, 39). Contributing to this effect is the KYRV motif (Fig. 2*B*; highlighted), a four amino acid motif at positions 66, 67, 69, and 76 in the α1 helix that is present in all HLA-C, but not in HLA-A or HLA-B (40). Because the gene conversion that created *B∗46:01* inserted the KYRV motif into the backbone of B∗46:01, we hypothesized that this may be the source of the difference in expression between B∗73:01 and B∗46:01. To test this, we examined the expression of swap mutants in HeLa cells in which the residues at positions 66, 67, and 69 (both alleles encode Val76) present in B∗46:01 were replaced with those found in highly expressed B∗73:01 (which lacks the KYR motif and encodes ICA at these residues; Fig. 2*B*) and compared them with their WT counterparts. Consistent with the results from 721.221 cells, we found that in HeLa cells, WT B∗73:01 is expressed at a significantly higher level than B∗46:01 (Fig. 2*C*). Further, we found that the B∗73:01-KYR mutant had significantly reduced expression and the B∗46:01-ICA mutant had significantly increased expression compared to their respective WTs (Fig. 2*C*). These results are consistent with the KYRV motif playing a significant role in determining the divergent levels of cell-surface expression between these two C1+ HLA-B alleles. Thus, while B∗73:01 and B∗46:01 both encode the C1 epitope and serve as ligands for KIR2DL3, they differ considerably in their α1 domain sequence, which not only correlates with their distinctive peptidomes, but also with their divergent cell-surface expression.

### Three dimensional structures of B∗73:01 reveal the molecular basis for peptide presentation

The exceptional number of polymorphisms between class I HLA alleles is what largely confers upon them their unique antigen presenting abilities. Amino acid diversity is most pronounced in the peptide binding groove, suggesting that different environments select for the presentation of different peptides. Since the peptidome of B∗73:01 is so unique, we sought to elucidate its three-dimensional structure to determine how its molecular architecture contributes to shaping its unusual peptide repertoire. Crystals of recombinant B∗73:01 alone were difficult to obtain, regardless of the synthetic peptide loaded. Thus, two alternative strategies were utilized to obtain high resolution protein structures. First, we generated high affinity synthetic antibody fragments (Fabs) to HLA-B∗73:01. Using this approach, we successfully generated seven Fabs that bound to B∗73:01, each with nanomolar affinity (Table S2, Fig. S6). Although these reagents did not help as chaperones for crystal formation, as hoped, we copurified two of them (B.1 and B.8) with recombinant B∗73:01 loaded with the nonamer peptide P2R (NRFLGDYVV) *via* a single-chain P2R-linker-β2m-linker-B∗73:01 construct (Fig. S7*A*) to generate a three-dimensional map by cryogenic-electron microscopy (cryo-EM) to a resolution of 3.1 Å (Fig. S7*B*, Table S3). Of note, the constant domain portion of FabB.1, which binds predominantly to B2m, is not visible in our map, likely due to a preferred orientation bias experienced during cryo-EM data collection (Fig. S8). Second, we were able to solve the crystal structure of B∗73:01 in complex with KIR2DL2, loaded with a 10mer peptide, NRFAGFGIGL (KP1), to 2.9 Å resolution (Table S4).

**Figure 3:**
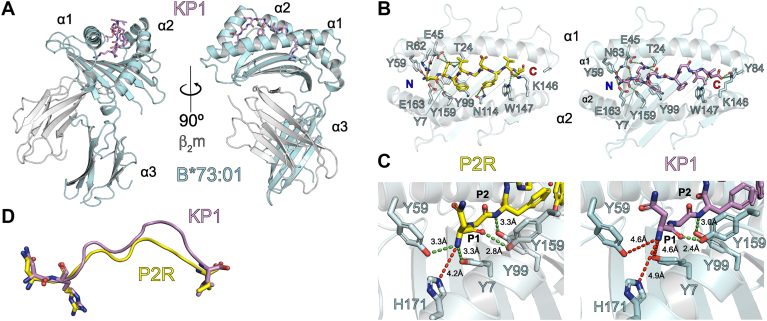
**High resolution protein structures of HLA-B∗73:01 bound to a 9mer and 10mer highlight flexibility within the peptide:MHC hydrogen-bonding network**. *A*, shown are two views of the structural model generated from the crystal structure of B∗73:01 with the KP1 peptide. The heavy chain of B∗73:01 is shown in *ribbon* format in *cyan*, β2m is shown in *ribbon* format in *gray* and the KP1 peptide is shown as *sticks* in *mauve*, with oxygens colored *red* and nitrogens, *blue*. *B*, comparison of the platform domains of B∗73:01 presenting the P2R (NRFLGDYVV) peptide, solved by cryo-EM structure (*left*) and that of B∗73:01 with the KP1 peptide (NRFAGFGIGL). The hydrogen bonding network between B∗73:01 and the P2R and KP1 peptides are indicated by *dashed green lines*. *C*, atomic distances between the amino terminus of each peptide and the corresponding residues forming the A pocket with distances in angstroms. Distances in *red* are outside the conventional hydrogen bonding cutoff of ∼3.5 Å. *D*, comparison of the backbones of the P2R and KP1 peptides from a superposition based on the B∗73:01 backbone. Peptide backbones are shown in cartoon format, colored as in (*B*) and (*C*). Side chains of the anchor residues are shown in *stick* format, with the coloring convention as in (A). Shown is the displacement distance, in Angstroms, of the P5 position of the peptides. MHC, major histocompatibility complex.

Our model of B∗73:01 generated using these two structures shows that it adopts an overall conformation similar to that of other class I HLA alleles (Fig. 3*A*) with adequate resolution to confidently place the peptides in both structures (Fig. S9). The backbone RMSD of the two structures of B∗73:01 was also quite small (0.746 Å over 274 Cα atoms), indicating that presentation of different peptides and receptor binding did not alter the three-dimensional structure of B∗73:01 significantly. Aside from hydrogen-bonding (h-bonding) between N114 of B∗73:01 and P7Y of the P2R peptide and a pi-stacking interaction between W147 of B∗73:01 and P6F of KP1, the networks of bonds (h-bonds, van der Waals (VDWs), and salt bridges, Table S5) that secure P2R and KP1 within the B∗73:01 binding groove are biased toward the N and C terminal ends of the peptide ligand (Fig. 3*B*), as is the case for most other class I HLA molecules. This bias is also consistent with trends seen in the peptide-binding motif where preferences are biased toward P2 and the C terminal position (PΩ) of the peptide (Fig. 1*C*). Unlike most other alleles, B∗73:01 has a slight preference for an asparagine at P1 of the peptide (Fig. 1*C*); both peptides in our B∗73:01 structures have this residue in the P1 position. In these two models, the asparagine side chain forms contacts with residues from the B∗73:01 α helices, engaging residues on either side of the peptide binding groove, namely N63 and E163 (Fig. 3, *B* and *C* left panels), whereas P1-N of P2R is further stabilized through a contact with R62 on B∗73:01. Thus, our structures have revealed the molecular basis of B∗73:01 peptide presentation of two peptides of differing length and demonstrate an alternative network of N terminal stabilizing interactions that function to accommodate peptides of different sequence and length.

Comparing the two structures of B∗73:01 with different peptides, we can also see examples of how peptides of different sequence and length are accommodated within the B∗73:01 groove. At the N and C termini of the peptide, almost all h-bonding contacts are conserved across the two structures within our resolution limits (Table S5). The only specific h-bonding contacts unique to each structure are those between the P2R peptide and Y59, R62, and N114 and the KP1 peptide and N63, N80, and Y84 (this side chain was not resolved in the P2R structure) (Fig. 3*C*). The P2R peptide is a 9mer, while KP1 is a 10mer, and the difference in lengths between these two peptides are accommodated through a displacement that is biased toward the C terminal end of the peptide. Indeed, the Ca backbone displacement of KP1 in relation to P2R at position 7 (glycine and tyrosine, respectively) is ∼4.4 Å (Fig. 3*D*). This tyrosine at P7 of the P2R peptide forms a unique contact with N114 of B∗73:01, further helping to anchor this in the groove (Fig. 3*B*).

Closer inspection of the hydrogen-bonding network between the backbone of the N terminus of the P2R and KP1 peptides and B∗73:01 revealed an adaptation that alters the canonical N terminal anchoring observed in most classical class I structures in a length-dependent fashion (Fig. 3*C*). In most class I HLA molecules, tyrosines at positions 7, 99, 159, and 171 coordinate the amine group of the N terminus of their peptide ligands (41). In B∗73:01, tyrosine 171 is replaced by a histidine; with the P2R peptide, h-bonding between the amino group and Y7 is maintained and even enhanced with Y59 (3.3 Å) (Fig. 3*B*). However, the N terminus of KP1 is positioned outside the distance of relevant hydrogen-bonding for these residues (Fig. 3*C*). In relation to the positioning of other canonical peptide N termini, the N terminus of KP1 is shifted up by an average of 1.64 Å relative to nonamers (Fig. S10*A*; left panel) and an average of 1.79 Å relative to other 10mers, an 11mer, and a 15mer (Fig. S10*A*; right panel). This contrasts with what is seen for the alleles B∗15:01, C∗01:02, and B∗27:05, where each allele has both Y7 and Y171 within reasonable distance to form hydrogen bonds with their respective peptide ligands (Fig. S10*B*, left panels). B∗14:02 and B∗51:01, in contrast, are some of only a small number of alleles that carry H171 instead of Y171. Interestingly, B∗14:02 shows a similar bonding network to B∗73:01 as well as a similar slight preference for asparagine or aspartic acid at P1 of its peptide ligands (Fig. S10*B*, right panel). Of note, although only one of multiple experimental structures was used for these analyses, by calculating distances between the Cαs of HLA platform residue 26 and peptide residue 1 (N-term distance) and the HLA platform residue 117 and the peptide omega residue (C-term distance) all for 674 peptide-HLA protein structures curated by the HLA3DB and the 2 B∗73:01 structures from this study we find that N-term distances for B∗73:01, B∗14:02, and B∗51:01—all alleles that encode H171—hold up across multiple structures, while the opposite is observed for HLA molecules that encode Y171 (Fig. S11). Previous studies investigating peptide binding to three alleles of HLA-B∗51 that carry either H171 or Y171 show that while the difference had minimal effect on peptide binding, it did have a functional effect on T cell responses (42, 43). Thus, the A-pocket of B∗73:01 carries H171 instead of Y171, which likely alters the conformation of bound peptides enough to affect T cell responses, but not its peptide repertoire.

Furthermore, our analysis of peptides of varying lengths presented by B∗73:01 discussed above showed conserved anchor residues near the N and C termini suggesting that as presented peptides increase in length, they likely bulge out from the binding groove to be accommodated. In our B∗73:01/KP1 structure, the conformation of the KP1 peptide (a 10mer) is displaced from the groove near the C terminus by an average of 3.11 Å in comparison to seven different nonamer peptides from other HLA structures (Fig. S10*A*, left panel). When compared to peptides of at least 10 amino acids in length, KP1 is further displaced in this location by an average of 3.57 Å (Fig. S10*A*, right panel). Together, these data suggest that the peptide binding mode of longer peptides presented by B∗73:01 differs from that of most other alleles in its anchoring at the N terminus within the A-pocket. Furthermore, B∗73:01 accommodates peptides of extreme length (11+ residues) through a bulged conformation toward the C terminal end of the peptide. It is of course also possible that some B∗73:01-presented peptides do not bulge, but rather extend through the open C terminal region of the peptide binding groove, especially since a number of the characterized peptides have a C-terminal Ala, which may not be an ideal anchor. Our analyses show that while we find that most peptides are thought to form a bulge, we do also find a significant proportion that are likely to extend through the C terminal end of the peptide-binding groove (Fig. S12). Lastly, while we also cannot rule out that the binding of KIR2DL2 has modified the conformation of the peptide in this structure, other structural comparisons between bound and unbound HLA molecules have shown little if any peptide movement when KIR bind. For example, a comparison of crystal structures of C∗07:02 loaded with the same peptide that crystallized alone, in complex with KIR2DL2, or in complex with KIR2DL3 shows minimal differences in the peptide backbone and side chains, suggesting that binding by KIR2DL2 likely does not alter the mode of presentation of KP1 presented by B∗73:01 (16) (Fig. S13).

### The B and F pockets of B∗73:01 illuminate constraints and flexibility in peptide binding

**Figure 4:**
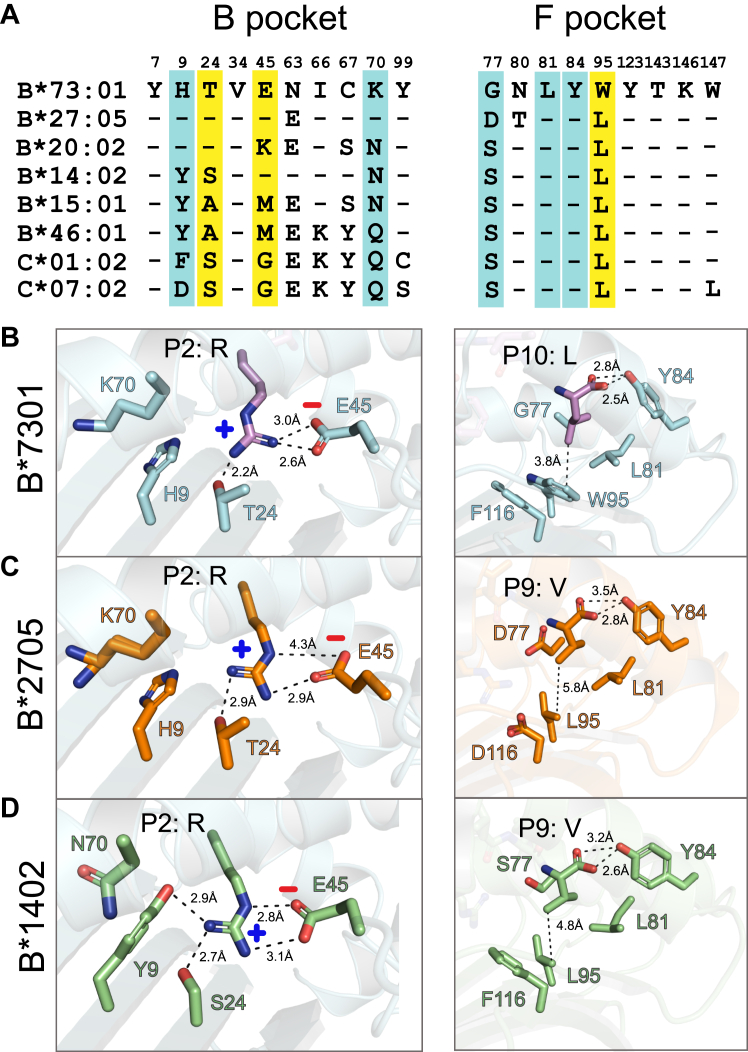
**Variation in the B and F pockets of B∗73:01 provide flexibility in accommodating diverse peptide anchor residues**. *A*, residues lining the B (*left*) and F (*right*) pockets of B∗73:01 and selected HLA alleles. Highlighted in *yellow* are key residues that engage the anchor residues shown in *panelsB*–*D*, supporting residues are highlighted in *cyan*. Contacts between key peptide anchor residues and pocket residues in the crystal structures of B∗73:01 (*B*), B∗27:01 (*C*) and B∗14:02 (*D*).

B∗73:01 is part of an ancient allelic lineage of HLA-B alleles, and the only member of this lineage in humans (6). The B pocket (the canonical pocket that binds the P2 of the peptide) of B∗73:01 uses very similar residues as those of B∗27:05 and B∗14:02 (Fig. 4*A*, left panel). An investigation of our structural model of B∗73:01 shows that T24 and E45 are likely very important for anchoring the P2 arginine within the B pocket (44) (Fig. 4, *A* and *B*, left panel, positions highlighted in yellow) for both the P2R and KP1 peptides. This is also similar to what is seen in B∗27:05 and B∗14:02 (Fig. 4, *C* and *D* left panel). Notably, a previous study performing quantitative peptide binding studies tested dozens of different peptides and their ability to stabilize the B∗73:01 trimeric complex in solution (25). Surprisingly, although the majority of peptides that bound most strongly contained an arginine at P2, the highest affinity binder contained a glutamic acid at P2. To reconcile this with our data, we noticed that B∗73:01 also carries H9 and K70 (Fig. 4*A*, left panel, positions highlighted in blue), two basic residues which are not involved in anchoring the peptides with a P2 arginine which may be recruited in stabilizing a P2 glutamic acid. Along these lines, B∗40:02 is a B allele that strongly prefers a glutamic acid anchor at P2 and also carries H9 and N70, both of which form hydrogen bonds with the peptide anchor residue (Figs. 4*A*, S14). Thus, B∗73:01 may, in rare cases, be able to adopt its peptide binding groove to present peptides anchored at P2 with either strongly basic or acidic properties.

Although B∗73:01 and B∗27:05 have highly correlated peptide binding motifs, they present very few of the same peptide sequences when purified from the same cell. They share similar peptide anchor strategies within their B pockets, yet they have evolved quite different F pockets. One F pocket residue that is quite rare and carried by B∗73:01 is W95, which only appears in roughly 12% of alleles in the set of alleles included in Sarkizova *et al.*, while roughly 43% contain a leucine at this position (Fig. 4*A*, right panel, highlighted in yellow). W95 likely contributes to the ability of B∗73:01 to present peptides that have more diversity at the C terminus than those presented by other HLAs likely due to its enhanced ability to shield small hydrophobic residues from water molecules, but also through CH/π interactions with proline residues at the C terminus of the peptide (another preferred residue for B∗73:01) (Fig. 4*B*, right panel). This combination of relative constraint within the B pocket combined with hydrophobic plasticity within the F pocket represents a unique tradeoff for B∗73:01 relative other alleles and contributes to its unique peptide repertoire, distinct from even B∗27:05.

### The complex structure of B∗73:01 and KIR2DL2 indicates the use of a unique docking angle

**Figure 5:**
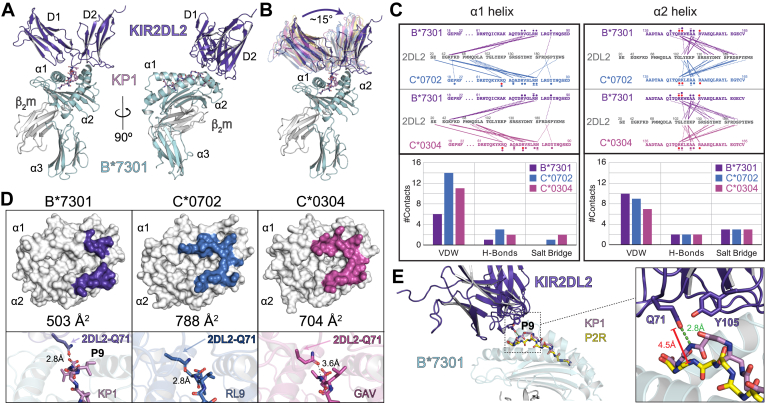
**The three dimensional structure of the complex between B∗73**:**01/KP1 and KIR2DL2**. *A*, two views, related by a 90 degree turn, of the complex; KIR2DL2 is shown in *ribbon* format, colored *dark purple*; HLA-B∗73:01 is colored as in Figure 3: heavy chain in *cyan*, β_2_m in *gray* and KP1 peptide in *mauve*. KIR2DL2 engages B∗73:01 directly over the F pocket. *B*, comparison of the KIR2DL2/B∗73:01-KP1 complex with that of representative KIR complexes with their cognate HLA. Shown are KIR2DL2 and KIR2DL3 engagement of HLA C∗07:02 (PDB ID: 6PA1 and 6PA2; semitransparent *marine blue* and semitransparent *magenta*, respectively) and KIR2DS2 engagement of HLAA∗11:01 (PDB ID: 4N8V, semitransparent *yellow*) (16, 45). HLAs were superimposed based on their Cα backbones. Shown is the angle differences between the KIR2DL2 docking on B∗73:01 in comparison with the KIRs docking on C∗07:02 and A∗11:01. *C*, contact analysis between the α helices of B∗73:01, C∗07:02, and C∗03:04 and KIR2DL2. Shown are the amino acid sequences of the α1 helix (*left*) and α2 helix (*right*) of the HLAs. *Lines* denote contacts between KIR2DL2 and these HLAs; *solid lines* are hydrogen bonds (<3.4 Å) and *dashed lines* are van der Waals (<4.5 Å) coloring is based on the relevant HLA. Symbols above and below the amino acid sequences indicate which type of interaction each residue contributes to: *circles* in backbone color: *VDW, lines*: h-bonds, *red circles*: electrostatic/salt bridges. Below the contact figure are *bar graphs* presenting the total number of contacts of each type per complex. *D*, the KIR2DL2 binding footprint on B∗73:01 (*dark purple*) *versus* C∗07:02 (*marine blue*, PDB ID: 6PA1) and C∗03:04 (*magenta*, PDB ID: 1EFX). HLAs are shown in surface representation colored *white* with the relevant KIR2DL2 buried surface area footprints colored as indicated above. Below each footprint representation is a zoomed view of the key KIR2DL2 residue Q71 in the interface with the respective HLAs. Q71 forms a hydrogen bond with the P8 position of the presented peptide backbone. In the complex structure with B∗73:01 (*left* panel), Q71 adopts a different conformation than it does when engaging C∗07:02 (*middle*) and C∗ 03:04 (*right*). *E*, comparison between the complex between KIR2DL2 and B∗73:01 with the KP1 peptide (10mer) and the modeled complex with the P2R peptide (9mer). Distance measurements are between the Q71 sidechain of 2DL2 and the P9 mainchain nitrogen of each peptide. KIR, killer-cell immunoglobulin-like receptor.

B∗73:01 and B∗46:01 are distinct from all other human B alleles in that they carry the C1 epitope (both V76 and N80 in the α1 helix) which confers reactivity to KIR2DL2/3. However, both alleles evolved their epitope independently (6, 15). Our complex crystal structure of B∗73:01/KP1 with KIR2DL2 provides new insight into how these B alleles engage with receptors that are predominantly restricted to HLA-C alleles, specifically compared to the complex structures of 2DL2 with HLA-C∗03:04 and HLA-C∗07:02. Our complex shows the general binding mode of KIR2DL2 onto B∗73:01 is grossly similar to that seen for other KIR complex structures published to date (Fig. 5*A*). That is, the KIR binds directly over the F pocket of the heavy chain, making no contact with b_2_m with the D1 domain generally making contacts with the α 1 helix of B∗73:01 and the D2 with the α 2 helix of the antigen binding region. Interestingly, however, when compared to other HLA/KIR2D complex structures (including KIR2DL2/C∗07:02, KIR2DL3/C∗03:04, and KIR2DS2/A∗11:01 (16, 45)) the docking angle of KIR2DL2 onto B∗73:01 is shifted ∼15° toward the α 2 helix (Fig. 5*B*). A contact map (Fig. 5*C*) and buried surface area analysis (Fig. 5*D*) of the intermolecular contacts between KIR2DL2 and B∗73:01 compared with those of the complexes of HLA- C∗07:02 and C∗03:04 with KIR2DL2, show that the D1 domain of KIR2DL2 uses considerably fewer contacts (6 VDW and 1 h-bond) to engage the α 1 helix of B∗73:01 than with the α 1 helices of HLA-C∗07:02 (14 VDW, 3 h-bond, and 1 salt bridge) and C∗03:04 (11 VDW, 2 h-bond, and 2 salt bridge). In contrast, the contacts between the D2 domain of KIR2DL2 and the α 2 helix of B∗73:01 have slightly more VDW contacts, and share the same number of h-bonds (2) and salt bridges (3) as with the complexes with C∗07:02 and C∗03:04. Together, this results in an overall smaller footprint surface area for B∗73:01 skewed toward the a2 helix, likely contributing to the “tilt” of KIR2DL2 on B∗73:01 relative to these other complexes.

A close-up view of the buried surface area between the KIR and presented peptide backbones shows that Q71 of KIR2DL2 has a direct h-bond contact with the main chain nitrogen of P9 of the KP1 peptide, either a result, or cause of, the skewed KIR binding (Fig. 5*D*). In other complex structures, Q71 remains closer to the α 1 helices of their corresponding HLA ligands. Because the KP1 peptide is likely presented this way regardless of binding to KIR2DL2, the engagement of Q71 with the backbone of KP1 likely further contributes to the unique docking angle of KIR2DL2 onto B∗73:01. Modeling of KIR 2DL2 onto the nonamer P2R peptide/B∗73:01 complex structure provides some insight into how peptide length and sequence may regulate KIR2DL2 engagement in a peptide specific way. Whereas KIR2DL2 *via* Q71 forms a strong h-bond with the KP1 backbone at position 9 (distance ∼ 2.8 Å), the backbone of P2R is ∼4.5 Å away from Q71 (Fig. 5*E*), too far a distance for effective h-bonding. KIR2DL2 also has an additional VDWs contact with KP1 *via* Y105, which is not resolved in our P2R peptide complex model. This complex structure also suggests that for KIR2DL2 engagement, peptides presented by B73 need to be within a narrow length range; too short, and Q71 cannot engage, whereas if they are too long (*i.e.* greater than 11mers), then they may disrupt KIR2DL2 binding by sterically inhibiting its binding over the F-pocket, as these peptides appear to be accommodated through “bulging out” near the C terminus.

## Discussion

Our results demonstrate that B∗73:01, an archaic allele that was likely reintroduced to the human population through introgression, presents a restricted repertoire of unusually long peptides with a preference for arginine at P2 and a hydrophobic residue at the C terminal position. Although other HLA alleles exist with these preferences (*i.e.* B∗27:05, B∗07:02, and B∗14:02, Fig. 1), the B∗73:01 peptide repertoire is highly unique, likely the result of a combination of factors, one of which is the ability to present longer peptides 11+ amino acids in length. Our structural analysis of B∗73:01 with the KP1 10-mer peptide provides a model for understanding the molecular basis for this repertoire selection including a shift in the N terminal peptide anchoring (Fig. 3, *C* and *D*) and a skewing of the peptide “bulge” toward the C terminus. Although all these factors combine to generate a binding cavity that is unlike that of many other HLAs, there are also other factors, such as availability of peptides or distinct interactions of B∗73:01 with the peptide-loading complex machinery that may also play a role in restricting this repertoire. In comparing the diversity of nonamers at each position more broadly, we noted that B∗27:05 has even greater restriction at P2 than B∗73:01. It is known that the KK10 peptide bound by B∗27:05 of HIV origin shows signatures of escape mutations at the P2 position, mutating an arginine anchor to a lysine in order to escape B∗27:05-mediated T-cell surveillance (46). More diversity in the B∗73:01 P2 anchor position may allow B∗73:01 to be more tolerant of such escape mutations relative to B∗27:05. Indeed, our data suggest that 1% of B∗73:01 presented peptides contain a lysine at P2, whereas only 0.2% of such peptides bind to B∗27:05.

Highly compact peptidomes, like that of B∗73:01, are thought to correlate with protection against closely related pathogens (36). To our knowledge, there are no epidemiologic studies that have correlated B∗73:01 with the development or outcome of infectious disease, likely due to the low frequency of B∗73:01 in the modern human population. However, HLA alleles that encode the C1 epitope, in combination with KIR2DL3, are known to provide protection against hepatitis C virus (18) (an RNA virus), providing some rational for B∗73:01 having this KIR specificity. Our data show that B∗73:01 is expressed at high levels on the cell surface, presumably much higher than HLA-C alleles, so B∗73:01 carrying of the C1 epitope may provide a strong signal to inhibitory KIR2DL2 and KIR2DL3. Our structural data provide insight into how an HLA-C biased KIR can engage with a C1 epitope carrying HLA-B allele; B∗73:01 engages KIR2DL2 with a skewed footprint, in part due to the engagement of the peptide backbone mediated by Q71 of 2DL2 (Fig. 5*C*). Since there are currently only seven known HLA:KIR complex strictures, and a thorough definition and investigation of KIR docking angles has not been performed, it remains difficult to speculate with any degree of certainty what the significance might be of bound peptide modulating KIR binding angle. However, it is clear that the residues of the bound peptide, particularly at positions P omega minus one and P omega minus two, have a strong influence over KIR binding as has been shown in previous studies. One interesting avenue to explore might be the effect of the KIR docking angle on signaling output. However, this would need to be carefully controlled such that similar affinity interactions with different docking angles are compared so as to properly control for both variables. However, one might imagine that different docking angles could potentially lead to differences in the ability of KIRs to cluster on the cell surface, a feature thought to be important for signal transduction. In addition, because peptides presented by B∗73:01 appear to selectively “bulge” out from the binding groove near the C terminus of the peptide, the longer the peptide the more likely it will sterically hinder the engagement of these KIR2D receptors. Presentation of longer peptides from pathogens might thus both recruit a T cell response (not investigated in this report) and disrupt any NK cell inhibition mediated through the C1 epitope of B∗73:01, leading to a more robust pathogen-specific response. Intriguingly, and in support of our hypothesis, a recent study suggests that RNA viruses were likely the major drivers of adaptive introgression of archaic alleles from Neanderthals to modern Europeans (47). Of course, as is the case with B∗27:05, it is also likely that predisposition to autoimmune disease may also result. This is one possible explanation for the paradoxical low incidence of B∗73:01 in the modern human population: as a result of its unique characteristics, it may have conferred exceptional protection against disease, but also exceptional predisposition to autoimmunity (presumably one under selective pressure) and thus fallen outside of the normal range for utility of an HLA molecule. Intriguingly, there is one case report in which B73 is strongly linked to pediatric mycosis fungicides (48) although the authors rightly conclude that the numbers are too low for the finding to be conclusive in any way.

In summary, our study shows that archaic B∗73:01 is a highly expressed HLA with a unique peptidome of unusually long length which may modulate its ability to engage with the KIR2Ds through its C1 epitope. These structural and functional characteristics distinguish B∗73:01 from other HLA class I alleles and likely provided early modern human migrants that inherited this allele from archaic humans with a selective advantage as they colonized Europe and Asia.

## Experimental procedures

### Peptidome sequencing and analysis

Isolation and analysis of peptides was performed as previously described (15). Briefly, 721.221 cells were transfected with constructs encoding a soluble form of HLA-B∗73:01. The sHLA-peptide complexes were affinity purified from the cell-supernatant on an anti-W6/32 Sepharose column, eluted and the bound peptides dissociated from the HLA by denaturation. Peptides were separated from the denatured proteins by ultrafiltration and separated into ∼40 fractions by reverse-phase high-performance liquid chromatography (HPLC) as described. Approximately 25% of each of the 40 HPLC fractions was injected into a nano-scale reverse phase liquid chromatography Eksigent nano-LC-4000 (Sciex) system. Column specifications, mobile phase solvents, and the elution gradient were as described (49). Eluted fractions were ionized using a NanoSpray III (Sciex) ion source into a Sciex TripleTOF 5600 mass spectrometer collecting LCMS spectra in data-dependent acquisition mode (n = 1 single injection across 40 different peptide-containing fractions). Sequences were assigned to spectra using PEAKS 7 at a 1% false discovery rate as described in (49).

PCA plots were generated using the PeptidePCA R package (https://github.com/ParhamLab/PeptidePCA). Briefly, for each amino acid in a peptide, four biochemical properties (molecular weight, hydropathy index, surface area, and isoelectric point) were determined. Thus 36 variables were generated for each nonamer peptide. Dimensionality reduction was performed on these variables for each peptide and colored by allele. Peptides of length 8 to 15 were extracted from the eluted ligand data for the four HLA class I alleles. A GibbsCluster analysis was performed on each data set to identify the majority binding motif and remove noise in the data.

### Integration with and analysis of Sarkizova *et al.* data

Curated peptide lists were downloaded from ftp://massive.ucsd.edu/MSV000084172/and compared directly to peptides eluted from B∗73:01. To calculate correlation coefficients in peptide space between all alleles, amino acid occurrences of all 20 amino acids were calculated for each position of all nonamers generating a 9 × 20 matrix for each allele. These matrices were then flattened into vectors of 180 integers for each allele and used to directly calculate the Pearson correlation coefficients between alleles of presented nonamers. For plotting, alleles were clustered by hierarchical clustering implemented by the ggcorrplot function of the ggcorrplot package in R.

To calculate correlation coefficients between alleles in pocket space, pseudosequences for each allele were used as described in (50). In brief, following a multiple sequence alignment, positions within the alignment that were 100% conserved between alleles were removed, leaving only variable aligned columns. These sequences were then used to calculate 61 different descriptors of their amino acid sequences using the aaDescriptors function of the Peptides package (https://pypi.org/project/peptides/) and default parameters. This generated a vector of 61 values for each allele which was then used to calculate Pearson correlation coefficients between all alleles. For plotting, alleles were clustered by hierarchical clustering implemented by the ggcorrplot function of the ggcorrplot package in R (https://cran.r-project.org/web/packages/ggcorrplot/readme/README.html). For plotting sequence logos, the R package ggseqlogo was used (51, 52). For calculating Shannon and Simpson diversity indices, the diversity function from the R package vegan (52) was used.

### Cell-surface expression experiments

Briefly, 721.221 cells, which lack endogenous HLA class I expression, were transfected with constructs encoding B∗73:01, B∗46:01, B∗15:01, or C∗01:02 and cultured as previously described. Cells expressing HLA class I were detected by flow cytometry (Accuri C6 cytometer, BD Biosciences). Expression levels of each allele was determined from the median fluorescence intensity of the W6/32 antibody bound to each positive staining cell. Three independent transfections with at least 50,000 cells each were performed for each allele tested.

We examined the cell-surface expression of WT and KYR/ICA mutant 3x FLAG-tagged B∗73:01 and B∗46:01 in HeLa cells. Recombinant complementary DNA encoding amino acids 1 to 338 of B∗73:01 and B∗46:01 with an N-terminal 3X FLAG-tag were manufactured by Genscript (Piscataway). Site-directed mutagenesis was performed with the QuikChange Kit (Stratagene), according to the manufacturer's instructions, to generate the two swap KYR/ICA mutants. HeLa cells were transfected with these constructs using the Fugene transfection reagent (Promega) and cultured as previously described (53). Cells expressing FLAG-tagged HLA class I were detected by flow cytometry (Accuri C6 cytometer, BD Biosciences). Expression levels of each allele or mutant were determined from the median fluorescence intensity of FITC-conjugated anti-FLAG antibody bound to each positive staining cell. Three independent transfections with at least 50,000 cells each were performed for each allele tested.

### Phage display selection protocol

To obtain high-affinity binders, five rounds of selection were conducted using the phage display selection protocol previously described (54). Biotinylated HLA-B∗73:01 was immobilized onto streptavidin-coated paramagnetic beads (Promega) for the selection process. In the initial round, 500 nM of HLA-B∗73:01 was immobilized on 200 μl SA magnetic beads and incubated with 1 ml phage library (10ˆ10 colony-forming units) for 1 h at room temperature with gentle agitation. The beads were subjected to three washing cycles to remove nonspecific phage, then introduced to log-phase *Escherichia coli* XL-1 blue cells and incubated for 20 min at room temperature. Subsequently, media containing 100 μg/ml ampicillin and 10ˆ9 p.f.u./ml of M13K07 helper phage (NEB) was added for overnight phage amplification at 37 °C. The amplified phage was precipitated in 20% PEG/2.5 M NaCl for 20 min on ice in preparation for subsequent rounds. Prior to each round, the phage pool underwent negative selection against empty paramagnetic beads for 30 min with shaking to eliminate nonspecific binders. The antigen concentration was systematically reduced from 500 nM to 10 nM from the first to the fifth round (second round: 250 nM, third round: 100 nM, fourth round: 50 nM, and fifth round: 10 nM). After phage binding, the beads were subjected to five washing rounds with 0.5% bovine serum albumin (BSA)/phosphate-buffered saline with Tween. Bound phages were eluted using 0.1 M glycine, pH 2.6, and neutralized with TRIS–HCl, pH 8. The phage eluate was then used for *E. coli* infection and phage amplification as previously described. Following the fourth and fifth rounds, infected cells were plated on ampicillin agar, and 192 colonies were selected to produce phage clones for single-point phage ELISA assay. Promising clones demonstrating high specificity were sequenced, reformatted into an RH2.2 expressing vector, and produced as previously described (55).

### Phage enzyme-linked immunosorbent assays

HLA-B∗73:01, at a concentration of 50 nM, was directly immobilized onto high-binding experimental wells (Greiner Bio-One) for a duration of 30 min. Subsequently, the wells were subjected to extensive blocking with 2% BSA for 1 h to minimize nonspecific binding. Following a 15-min incubation period with phage, the wells underwent a rigorous washing protocol, consisting of three cycles with 0.5% BSA/phosphate-buffered saline with Tween. The wells were then incubated with Protein L-HRP (Thermo Fisher Scientific) at a 1:5000 dilution in Hepes-buffered saline with Tween for 20 min. After another thorough washing step, the plates were developed using TMB substrate (Thermo Fisher Scientific). The enzymatic reaction was quenched with 10% H3PO4, and the absorbance was quantified spectrophotometrically at 450 nm (A450).

### Structural determination and analysis by electron microscopy

#### Sample preparation and data acquisition

Quantifoil (R1.2/1.3, 200 mesh) gold grids (Ted Pella) were glow-discharged for 10 s at 20 W using the Solarus 950 Plasma Cleaner System (Gatan). Fluorinated octyl maltoside (Anatrace) was added to the complex of single-chain P2R-linker-β2m-linker-B∗73:01 MHC along with both B.1, and B.8 Fabs to a final concentration of about 0.07 mM before plunge freezing in Hepes-buffered saline with a pH of 7.2. Sample (3 μl at 0.6 mg ml-1) was applied to grids, which were blotted for one second at blot force two, using a Vitrobot Mark IV (Thermo Fisher Scientific) maintained at 8 °C in a 100% humidity environment, and plunge frozen into liquid ethane. Data were collected using a 300 kV Titan Krios G3i (Thermo Fisher Scientific) microscope equipped with a K3 detector (Gatan). We used a nominal magnification of 810,00x, translating to a pixel size 0.5325 Å in the raw micrographs. EPU software was set to automated acquisition mode and collected 5291 movies, each with 50 frames, subject to a total dose of 60 e−/Å^2^.

#### Image processing

Movies were motion-corrected and dose-weighted using MotionCor2 (56) in the RELION-3.1.3 (57) wrapper, with data binned 2X to 1.065 Å/pixel. Micrographs were imported into cryoSPARC v3.3.1 (58) and subjected to CTF Patch Estimation. Particle coordinates were identified by reference-free “blob” particle picking. A 256 pixel box was used to extract 4X binned (2.13 Å/pixel) particles, which were then sorted into 50 2D classes, several of which were selected as templates. Template particle picking yielded an initial set of 4,007,333 particles, which were culled to 1,531,860 following an additional round of 2D classification and used to generate three *ab initio* 3D volumes. These volumes were used as templates to sort particles into three heterogeneous refinement classes. Particles from the two best classes were subjected to another round of 2D classification, from which four 2D classes were chosen for a new round of template particle picking.

Subsequently, 4,844,663 particles were extracted and sorted into 50 classes in two consecutive rounds of 2D classification. A total of 2,217,300 particles were extracted in 256 pixel boxes (at 2.13 Å/pixel) and selected for *ab initio* reconstruction into four classes. The two highest-resolution 3D volumes, as well as one junk (“garbage collector”) class were selected as initial models for heterogeneous refinement. All three output volumes, as well as 1,141,440 particles associated with the highest-resolution class, were selected for an additional round of heterogeneous refinement. The highest-resolution refinement map and 732,360 associated particles were then selected as inputs for homogeneous refinement. Though estimated to have 3.2 Å resolution by cryoSPARC, the homogeneous refinement map exhibited prominent streaking in underrepresented orientations. The map and particles were therefore exported to RELION-3.1.3 for further processing.

Following 2D classification of the exported particles, 682,726 were subjected to a round of high-iteration 3D classification in which they were aligned to the 3D volume obtained from cryoSPARC's homogeneous refinement and sorted into two classes. The angular sampling interval was progressively lowered every 50 iterations. The final set of 502,247 particles underwent several rounds of 3D autorefinement, as well as CTF refinement. A solvent mask was generated for the highest-resolution autorefined map, which then underwent B-factor sharpening to yield the final reconstruction.

#### Refinement

Initial models used included the crystal structure of B∗73:01 determined in this study (Protein Data Bank (PDB): 8TMU) and AlphaFold2 (59) models generated for B.1 and B.8 Fabs using their respective primary amino acid sequences. Initial model docking into the highest-resolution refined map was done manually using ChimeraX (60) version 1.5 and the *fitmap* command. Additional rounds of real-space refinement were performed using PHENIX (61) version 1.20.1 to 4487-000.

### Crystal structure determination and analysis

Soluble B∗73:01 heavy chain (residues 1–276) and β_2_M light chain constructs were expressed in *E. coli* and refolded from inclusion body preps with respective peptides. A soluble KIR2DL2∗001 construct (residues 1–225 of the mature protein), covalently linked to an N-terminal 8x HisTag, separated by a 3C protease cleavage site, was expressed in BTI-Tn-5B1-4 insect cells (High Five) cells cultured in Insect-XPRESS Protein-free Insect Cell Medium (Lonza). BestBac 2.0 linearized DNA (Expression Systems) was used to transfect Sf9 cells to generate a P1 viral stock. Virus was then amplified by shaking culture in Sf9 cells. Following Ni-NTA affinity purification, KIR2DL2 was treated for 2 h at 37 °C with EndoF (made recombinantly in the Adams Lab) prior to another round of Ni-NTA affinity purification to remove any non-HisTagged EndoF. The 8xHisTag was removed using 3C protease (made recombinantly in the Adams Lab). Recombinant B∗73:01 and KIR2DL2 was purified separately by SEC into 10 mM Hepes, 150 mM NaCl, pH 7.2 (Hepes-buffered saline), mixed at a 1:1 M ratio, and concentrated to 6 to 10 mg/ml. Crystals were initially grown in less than 24 h in mother liquor containing Tris–HCl pH 8.5 and 25% PEG 8000. These crystals were then crushed and used as microseeds to grow a larger crystal in mother liquor containing Tris–HCl pH 8.75 and 20% PEG 8000, which took 1 to 2 weeks to grow.

CCP4, Coot, and PHENIX software suites were used for molecular replacement and refinement. PyMol was used to make figures and measure distances between atoms (57, 58, 59, 62, 63, 64, 65).

## Data availability

The refined structure coordinates have been deposited in the Protein Data Bank (www.rcsb.org) with accession code of 8TMU for the crystal structure. The cryo-EM structure was deposited with PDB accession code 8TNJ, and an EMDB number (www.ebi.ac.uk/emdb/) of EMD-41418.

## Supporting information

This article contains supporting information.

## Conflict of interest

The authors declare that they have no conflicts of interest with the contents of this article.

## Supporting information

Supporting Information

Peaks

## References

[1] Green R.E., Krause J., Briggs A.W., Maricic T., Stenzel U., Kircher M. (2010). A draft sequence of the Neandertal genome. Science.

[2] Meyer M., Kircher M., Gansauge M.-T., Li H., Racimo F., Mallick S. (2012). A high-coverage genome sequence from an archaic Denisovan individual. Science.

[3] Prufer K., Racimo F., Patterson N., Jay F., Sankararaman S., Sawyer S. (2014). The complete genome sequence of a Neanderthal from the Altai mountains. Nature.

[4] Reich D., Green R.E., Kircher M., Krause J., Patterson N., Durand E.Y. (2010). Genetic history of an archaic hominin group from Denisova Cave in Siberia. Nature.

[5] Juric I., Aeschbacher S., Coop G. (2016). The strength of selection against neanderthal introgression. PLoS Genet..

[6] Abi-Rached L., Jobin M.J., Kulkarni S., McWhinnie A., Dalva K., Gragert L. (2011). The shaping of modern human immune systems by multiregional admixture with archaic humans. Science.

[7] Vernot B., Akey J.M. (2014). Resurrecting surviving neandertal lineages from modern human genomes. Science.

[8] Dannemann M., Andrés A.M., Kelso J. (2016). Introgression of Neandertal- and Denisovan-like haplotypes contributes to adaptive variation in human toll-like receptors. Am. J. Hum. Genet..

[9] Henn B.M., Cavalli-Sforza L.L., Feldman M.W. (2012). The great human expansion. Proc. Natl. Acad. Sci. U. S. A.

[10] Parham P., Arnett K.L., Adams E.J., Barber L.D., Domena J.D., Stewart D. (1994). The HLA-B73 antigen has a most unusual structure that defines a second lineage of HLA-B alleles. Tissue Antigens.

[11] Moesta A.K., Norman P.J., Yawata M., Yawata N., Gleimer M., Parham P. (2008). Synergistic polymorphism at two positions distal to the ligand-binding site makes KIR2DL2 a stronger receptor for HLA-C than KIR2DL3. J. Immunol..

[12] Hilton H.G., Vago L., Older Aguilar A.M., Moesta A.K., Graef T., Abi-Rached L. (2012). Mutation at positively selected positions in the binding site for HLA-C shows that KIR2DL1 is a more refined but less adaptable NK cell receptor than KIR2DL3. J. Immunol..

[13] Barker D.J., Maccari G., Georgiou X., Cooper M.A., Flicek P., Robinson J. (2023). The IPD-IMGT/HLA database. Nucleic Acids Res..

[14] Barber L.D., Percival L., Valiante N.M., Chen L., Lee C., Gumperz J.E. (1996). The inter-locus recombinant HLA-B∗4601 has high selectivity in peptide binding and functions characteristic of HLA-C. J. Exp. Med..

[15] Hilton H.G., McMurtrey C.P., Han A.S., Djaoud Z., Guethlein L.A., Blokhuis J.H. (2017). The intergenic recombinant HLA-B∗46:01 has a distinctive peptidome that includes KIR2DL3 ligands. Cell Rep..

[16] Moradi S., Stankovic S., O'Connor G.M., Pymm P., MacLachlan B.J., Faoro C. (2021). Structural plasticity of KIR2DL2 and KIR2DL3 enables altered docking geometries atop HLA-C. Nat. Commun..

[17] Moesta A.K., Parham P. (2012). Diverse functionality among human NK cell receptors for the C1 epitope of HLA-C: KIR2DS2, KIR2DL2, and KIR2DL3. Front. Immunol..

[18] Khakoo S.I., Thio C.L., Martin M.P., Brooks C.R., Gao X., Astemborski J. (2004). HLA and NK cell inhibitory receptor genes in resolving hepatitis C virus infection. Science.

[19] Seich Al Basatena N.-K., Macnamara A., Vine A.M., Thio C.L., Astemborski J., Usuku K. (2011). KIR2DL2 enhances protective and detrimental HLA class I-mediated immunity in chronic viral infection. PLoS Pathog..

[20] Li S.S., Hickey A., Shangguan S., Ehrenberg P.K., Geretz A., Butler L. (2022). HLA-B∗46 associates with rapid HIV disease progression in Asian cohorts and prominent differences in NK cell phenotype. Cell Host Microbe.

[21] Schlosstein L., Terasaki P.I., Bluestone R., Pearson C.M. (1973). High association of an HL-A antigen, W27, with ankylosing spondylitis. N. Engl. J. Med..

[22] Migueles S.A., Sabbaghian M.S., Shupert W.L., Bettinotti M.P., Marincola F.M., Martino L. (2000). HLA B∗5701 is highly associated with restriction of virus replication in a subgroup of HIV-infected long term nonprogressors. Proc. Natl. Acad. Sci. U. S. A.

[23] Ombrello M.J., Kirino Y., de Bakker P.I., Gul A., Kastner D.L., Remmers E.F. (2014). Behcet disease-associated MHC class I residues implicate antigen binding and regulation of cell-mediated cytotoxicity. Proc. Natl. Acad. Sci. U. S. A.

[24] Barber L.D., Percival L., Parham P. (1996). Characterization of the peptide-binding specificity of HLA-B∗7301. Tissue Antigens.

[25] Sylvester-Hvid C., Kristensen N., Blicher T., Ferré H., Lauemøller S.L., Wolf X.A. (2002). Establishment of a quantitative ELISA capable of determining peptide - MHC class I interaction. Tissue Antigens.

[26] Shimizu Y., DeMars R. (1989). Production of human cells expressing individual transferred HLA-A,-B,-C genes using an HLA-A,-B,-C null human cell line. J. Immunol..

[27] Knierman M.D., Lannan M.B., Spindler L.J., McMillian C.L., Konrad R.J., Siegel R.W. (2020). The human leukocyte antigen class II immunopeptidome of the SARS-CoV-2 spike glycoprotein. Cell Rep.

[28] Reynisson B., Alvarez B., Paul S., Peters B., Nielsen M. (2020). NetMHCpan-4.1 and NetMHCIIpan-4.0: improved predictions of MHC antigen presentation by concurrent motif deconvolution and integration of MS MHC eluted ligand data. Nucleic Acids Res..

[29] Andreatta M., Lund O., Nielsen M. (2013). Simultaneous alignment and clustering of peptide data using a Gibbs sampling approach. Bioinformatics.

[30] Andreatta M., Alvarez B., Nielsen M. (2017). GibbsCluster: unsupervised clustering and alignment of peptide sequences. Nucleic Acids Res..

[31] Sesma L., Montserrat V., Lamas J.R., Marina A., Vázquez J., López de Castro J.A. (2002). The peptide repertoires of HLA-B27 subtypes differentially associated to spondyloarthropathy (B∗2704 and B∗2706) differ by specific changes at three anchor positions. J. Biol. Chem..

[32] Sarkizova S., Klaeger S., Le P.M., Li L.W., Oliveira G., Keshishian H. (2020). A large peptidome dataset improves HLA class I epitope prediction across most of the human population. Nat. Biotechnol..

[33] Scull K.E., Dudek N.L., Corbett A.J., Ramarathinam S.H., Gorasia D.G., Williamson N.A. (2012). Secreted HLA recapitulates the immunopeptidome and allows in-depth coverage of HLA A∗02:01 ligands. Mol. Immunol..

[34] Kaufman J. (2018). Generalists and specialists: a new view of how MHC class I molecules fight infectious pathogens. Trends Immunol..

[35] Shannon C.E. (1948). A mathematical theory of communication. Bell Syst. Tech. J..

[36] Chappell P., Meziane E.K., Harrison M., Magiera Ł., Hermann C., Mears L. (2015). Expression levels of MHC class I molecules are inversely correlated with promiscuity of peptide binding. Elife.

[37] Parham P., Barnstable C.J., Bodmer W.F. (1979). Use of a monoclonal antibody (W6/32) in structural studies of HLA-A,B,C, antigens. J. Immunol..

[38] McCutcheon J.A., Gumperz J., Smith K.D., Lutz C.T., Parham P. (1995). Low HLA-C expression at cell surfaces correlates with increased turnover of heavy chain mRNA. J. Exp. Med..

[39] Neisig A., Melief C.J., Neefjes J. (1998). Reduced cell surface expression of HLA-C molecules correlates with restricted peptide binding and stable TAP interaction. J. Immunol..

[40] Sibilio L., Martayan A., Setini A., Monaco E.L., Tremante E., Butler R.H. (2008). A single bottleneck in HLA-C assembly. J. Biol. Chem..

[41] Latron F., Pazmany L., Morrison J., Moots R., Saper M.A., McMichael A. (1992). A critical role for conserved residues in the cleft of HLA-A2 in presentation of a nonapeptide to T cells. Science.

[42] Kawaguchi G., Hildebrand W.H., Hiraiwa M., Karaki S., Nagao T., Akiyama N. (1992). Two subtypes of HLA-B51 differing by substitution at position 171 of the alpha 2 helix. Immunogenetics.

[43] Kikuchi A., Sakaguchi T., Miwa K., Takamiya Y., Rammensee H.G., Kaneko Y. (1996). Binding of nonamer peptides to three HLA-B51 molecules which differ by a single amino acid substitution in the A-pocket. Immunogenetics.

[44] Nguyen A.T., Szeto C., Gras S. (2021). The pockets guide to HLA class I molecules. Biochem. Soc. Trans..

[45] Liu J., Xiao Z., Ko H.L., Shen M., Ren E.C. (2014). Activating killer cell immunoglobulin-like receptor 2DS2 binds to HLA-A∗11. Proc. Natl. Acad. Sci. U. S. A.

[46] Goulder P.J., Brander C., Tang Y., Tremblay C., Colbert R.A., Addo M.M. (2001). Evolution and transmission of stable CTL escape mutations in HIV infection. Nature.

[47] Enard D., Petrov D.A. (2018). Evidence that RNA viruses drove adaptive introgression between neanderthals and modern humans. Cell.

[48] Reiter O., Ben Amitai D., Amitay-Laish I., Israeli M., Pavlovsky L., Hodak E. (2017). Pediatric mycosis fungoides: a study of the human leukocyte antigen system among Israeli Jewish patients. Arch. Dermatol. Res..

[49] Trolle T., McMurtrey C.P., Sidney J., Bardet W., Osborn S.C., Kaever T. (2016). The length distribution of class I-Restricted T cell epitopes is determined by both peptide supply and MHC allele-specific binding preference. J. Immunol..

[50] O'Donnell T.J., Rubinsteyn A., Laserson U. (2020). MHCflurry 2.0: improved pan-allele prediction of MHC class I-Presented peptides by incorporating antigen processing. Cell Syst.

[51] Wagih O. (2017). Ggseqlogo: a versatile R package for drawing sequence logos. Bioinformatics.

[52] Oksanen J., Simpson G.L., Blanchet F.G., Kindt R., Legendre P., Minchin P.R. (2022). Vegan: Community Ecology Package. https://cran.r-project.org/package=vegan.

[53] Hilton H.G., Norman P.J., Nemat-Gorgani N., Goyos A., Hollenbach J.A., Henn B.M. (2015). Loss and gain of natural killer cell receptor function in an African hunter-gatherer population. PLoS Genet..

[54] Slezak T., Kossiakoff A.A. (2021). Engineered ultra-high affinity synthetic antibodies for SARS-CoV-2 neutralization and detection. J. Mol. Biol..

[55] Slezak T., O'Leary K.M., Li J., Rohaim A., Davydova E.K., Kossiakoff A.A. (2024). Engineered Protein-G variants for plug-and-play applications. bioRxiv.

[56] Zheng S.Q., Palovcak E., Armache J.P., Verba K.A., Cheng Y., Agard D.A. (2017). MotionCor2: anisotropic correction of beam-induced motion for improved cryo-electron microscopy. Nat. Methods.

[57] Zivanov J., Nakane T., Forsberg B.O., Kimanius D., Hagen W.J., Lindahl E. (2018). New tools for automated high-resolution cryo-EM structure determination in RELION-3. Elife.

[58] Punjani A., Rubinstein J.L., Fleet D.J., Brubaker M.A. (2017). cryoSPARC: algorithms for rapid unsupervised cryo-EM structure determination. Nat. Methods.

[59] Jumper J., Evans R., Pritzel A., Green T., Figurnov M., Ronneberger O. (2021). Highly accurate protein structure prediction with AlphaFold. Nature.

[60] Goddard T.D., Huang C.C., Meng E.C., Pettersen E.F., Couch G.S., Morris J.H. (2018). UCSF ChimeraX: meeting modern challenges in visualization and analysis. Protein Sci..

[61] Liebschner D., Afonine P.V., Baker M.L., Bunkoczi G., Chen V.B., Croll T.I. (2019). Macromolecular structure determination using X-rays, neutrons and electrons: recent developments in Phenix. Acta Crystallogr. D Struct. Biol..

[62] Pettersen E.F., Goddard T.D., Huang C.C., Meng E.C., Couch G.S., Croll T.I. (2021). UCSF ChimeraX: structure visualization for researchers, educators, and developers. Protein Sci..

[63] Liebschner D., Afonine P.V., Baker M.L., Bunkóczi G., Chen V.B., Croll T.I. (2019). Macromolecular structure determination using X-rays, neutrons and electrons: recent developments in Phenix. Acta Crystallogr. D Struct. Biol..

[64] Agirre J., Atanasova M., Bagdonas H., Ballard C.B., Baslé A., Beilsten-Edmands J. (2023). The CCP4 suite: integrative software for macromolecular crystallography. Acta Crystallogr. D Struct. Biol..

[65] Fairhead M., Howarth M., Gautier A., Hinner M.J. (2015). Methods and Protocols.

