# Supplementary material for: Molecular characterization of the archaic HLA-B∗73:01 allele reveals presentation of a unique peptidome and skewed engagement by KIR2DL2
Source: J Biol Chem. 2025 Jul 30;301(9):110542. doi: 10.1016/j.jbc.2025.110542 (PMC12450638; doi:10.1016/j.jbc.2025.110542)
Supplement: Supporting Information [file mmc1.pdf]

Supplementary Materials

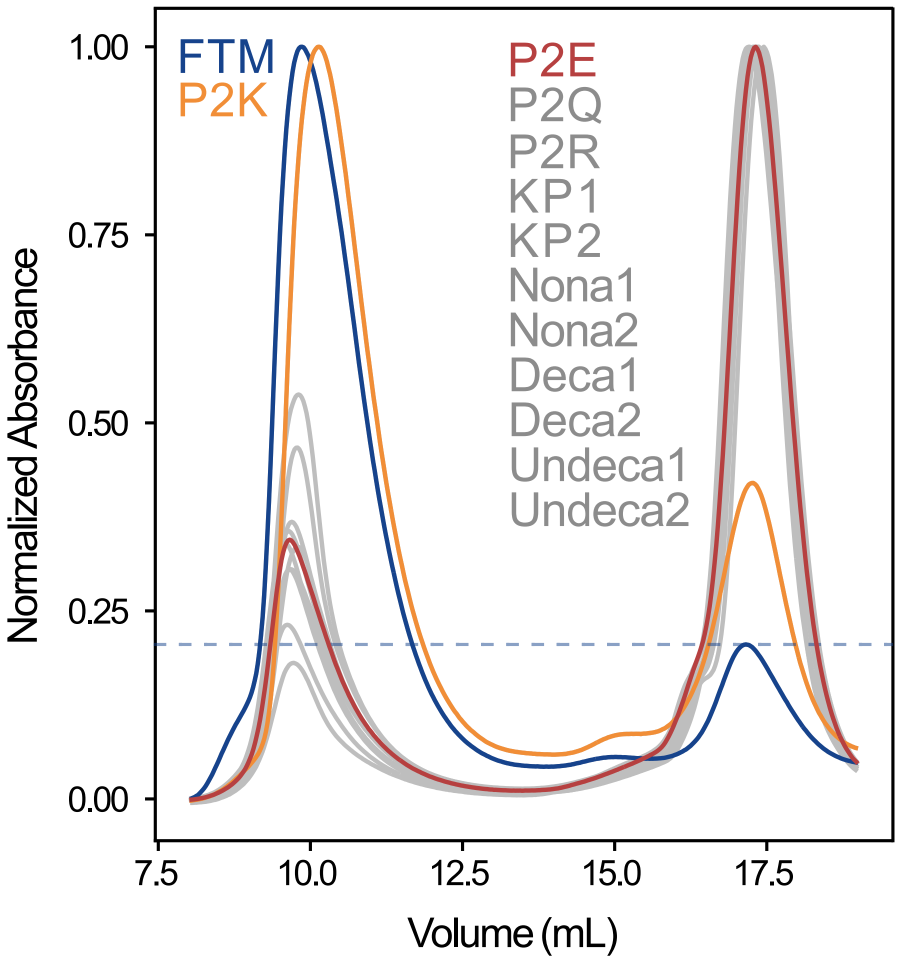

Figure S1. Size exclusion chromatograms of HLA-B*7301 refolded with indicated peptides. The Absorbance peak eluting at 10 mL is considered the aggregate peak while the peak eluting at 17.5 mL is considered the soluble monomeric peak. The FTM peptide is known to bind with high affinity to HLA-B*4601 and was used as a negative control of this experiment to indicate successful refolding.

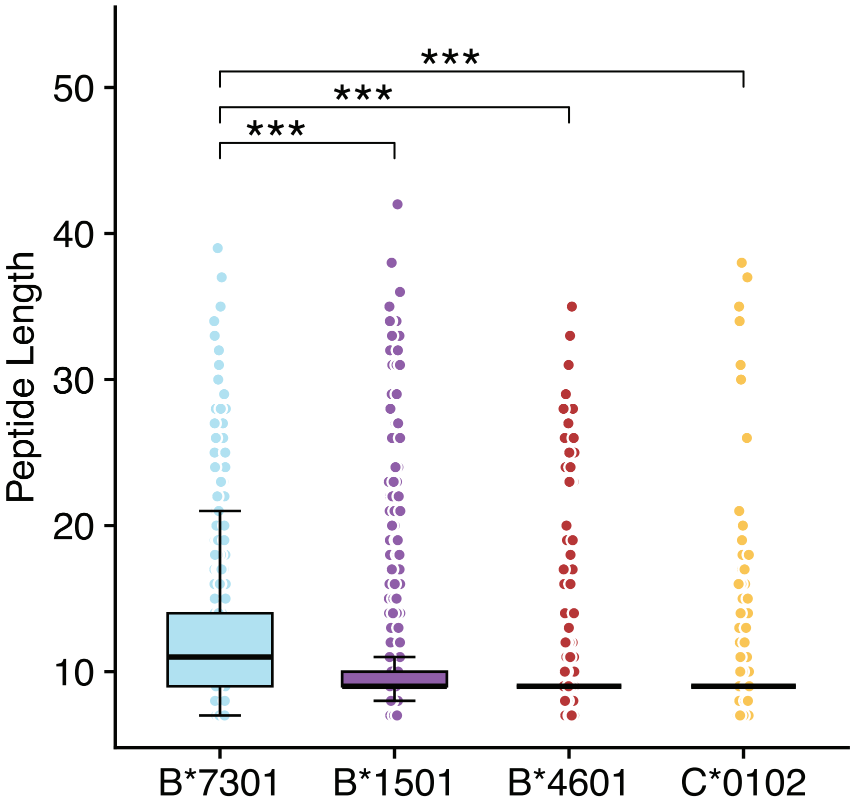

Figure S2: Box plots of eluted peptide lengths for peptides eluted from HLA-B*7301, HLA-B*1501, HLA-B*4601, and HLA-C*0102. Mean peptide lengths were compared using a Student's t-test. *** is equivalent to a p-value of less than 0.001.

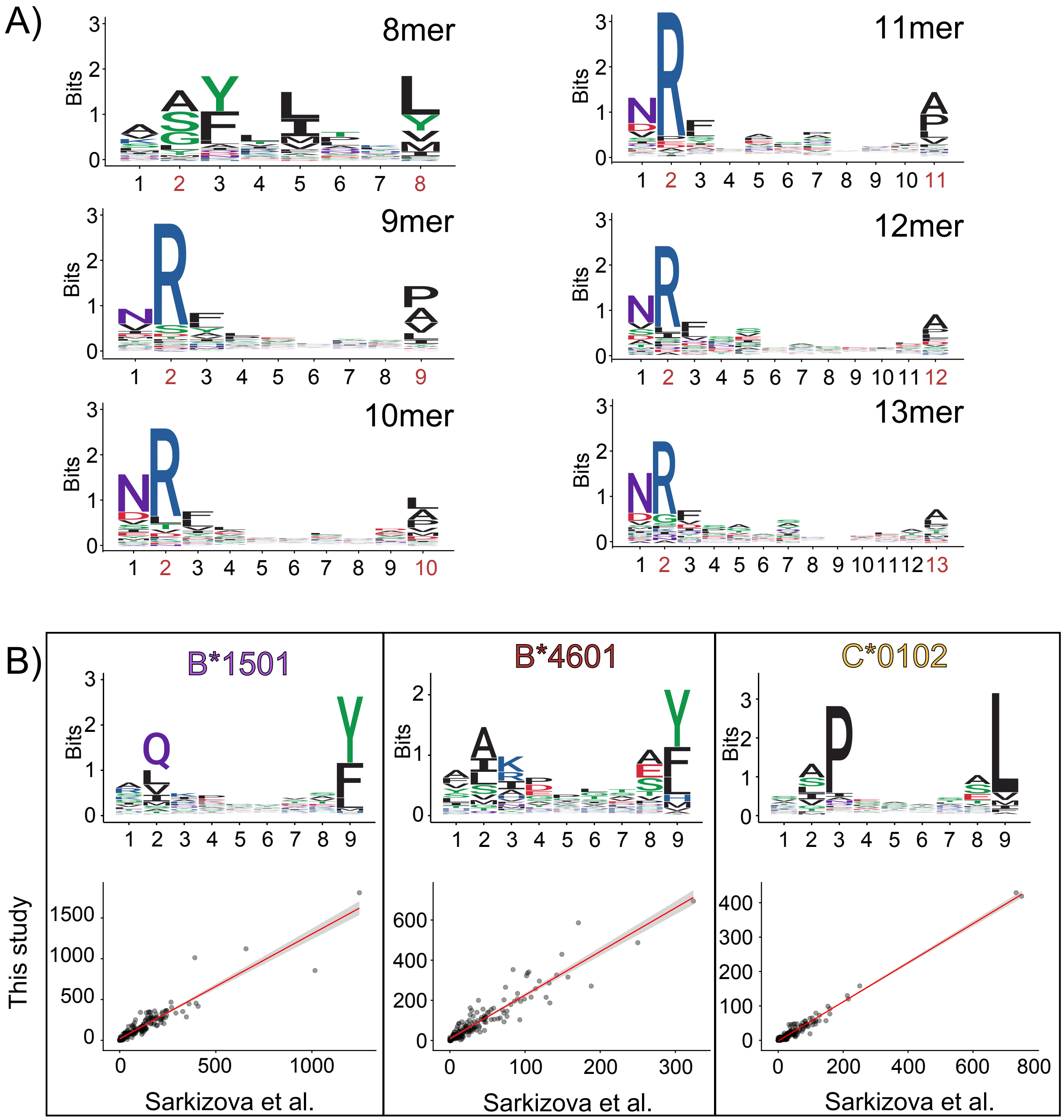

Figure S3: A) Sequence logos of peptides eluted from B*7301 stratified by length including peptides of length 8 (8mer) to length 13 (13mer). The colors of each amino acid correspond to their biochemical characteristics: acidic (red), basic (blue), hydrophobic (black) and polar (green). **B)** The 9mer peptide binding motif profiles (upper panels) of B*1501, B*4601, and C*0102 from Sarkizova et al. correlate positively (Pearson) with data generated in our previous study as shown through scatterplots (lower panels) of total bound peptides by alleles in this study plotted across the cell-surface expression values of the same alleles as expressed on 721.221 cells taken from Bashirova et al. 2020. Correlations were calculated using the *cor.test()* function in R and plotted using the *lm()* function.

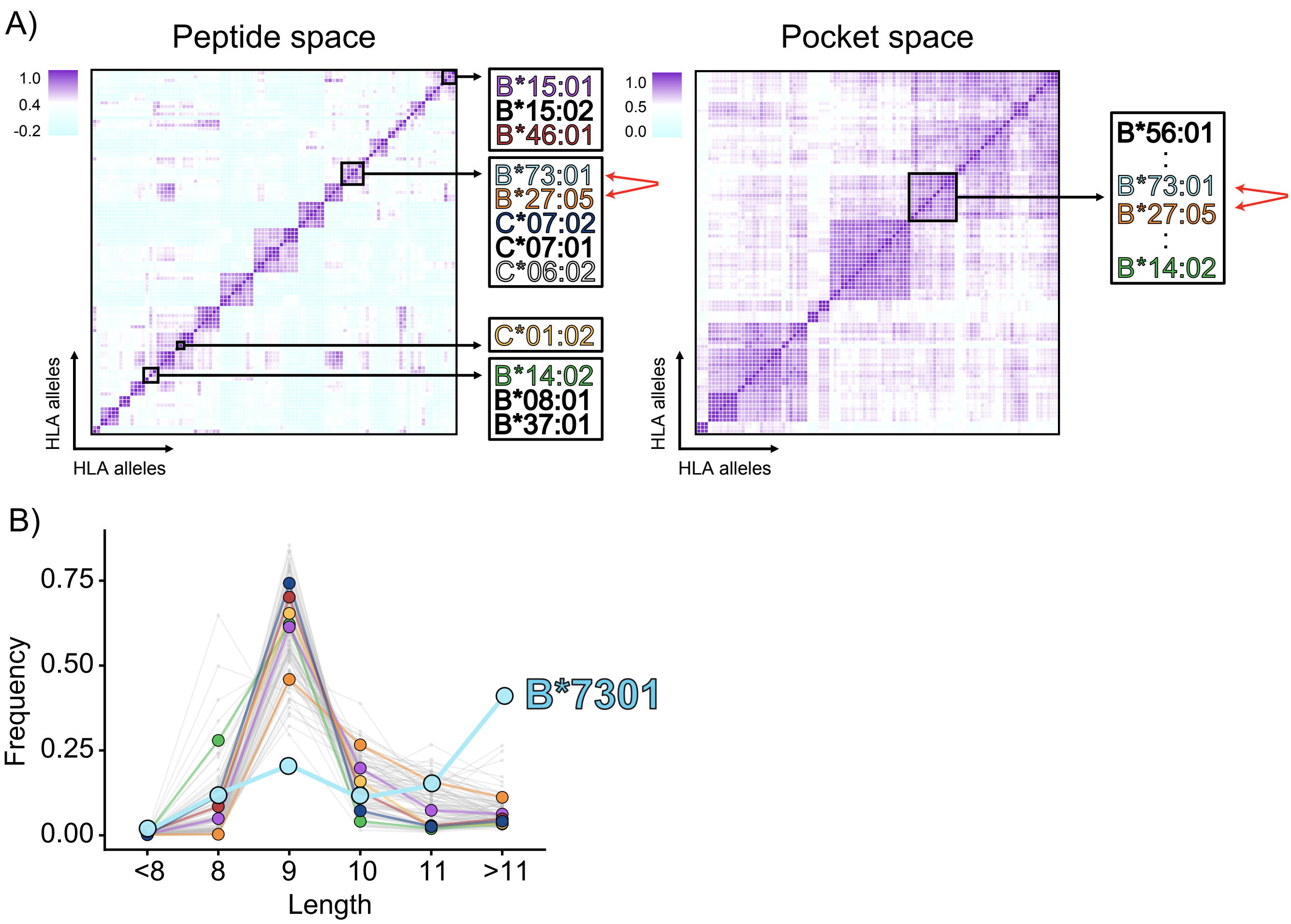

Figure S4: A) Heatmaps were generated using data from Sarkizova et al. was combined with eluted peptides from HLA-B*73:01 and representative peptides known to bind HLA-E (IEDB references 1037304, 1032571, and 1004539) and then correlated (Pearson) with other peptidomes (left; peptide space) and other MHC pocket residues (right; pocket space), then clustered based on similarity to highly alleles with similar peptide binding motifs and/or similar binding grooves. Alleles with similar peptide repertoires and/or similar binding grooves are depicted on the right. **B)** Length distributions of different alleles from Sarkizova et al. grouped into peptides less than 8, 8, 9, 10, 11, or more than 11 residues in length and including B*7301, shown in cyan.

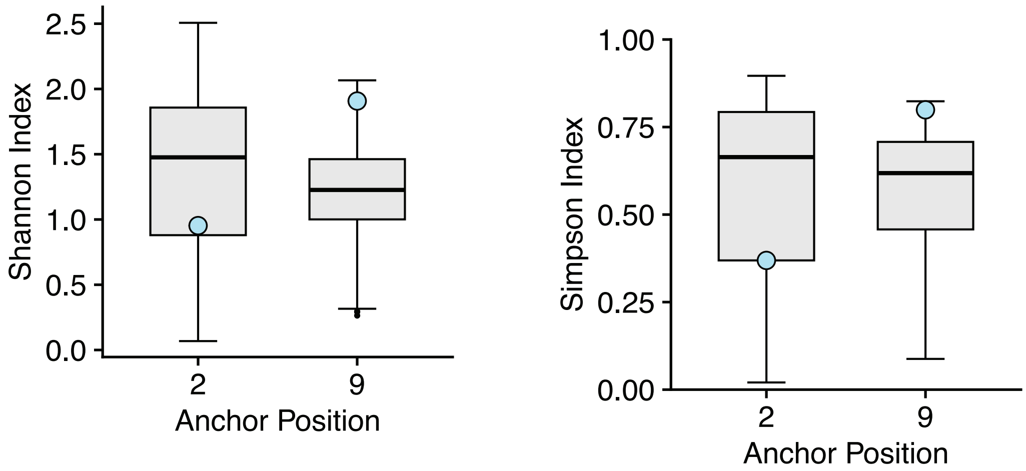

Figure S5: Box plots of Shannon and Simpson diversity indices for anchor positions P2 and P9 (i.e. P). A cyan data point is added to show where HLA-B*7301 falls on this distribution relative to other HLA alleles include in the analysis. Results show that HLA-B*7301 is not a statistical outlier relative to other HLA alleles in the number of residues anchored at positions 2 or 9.

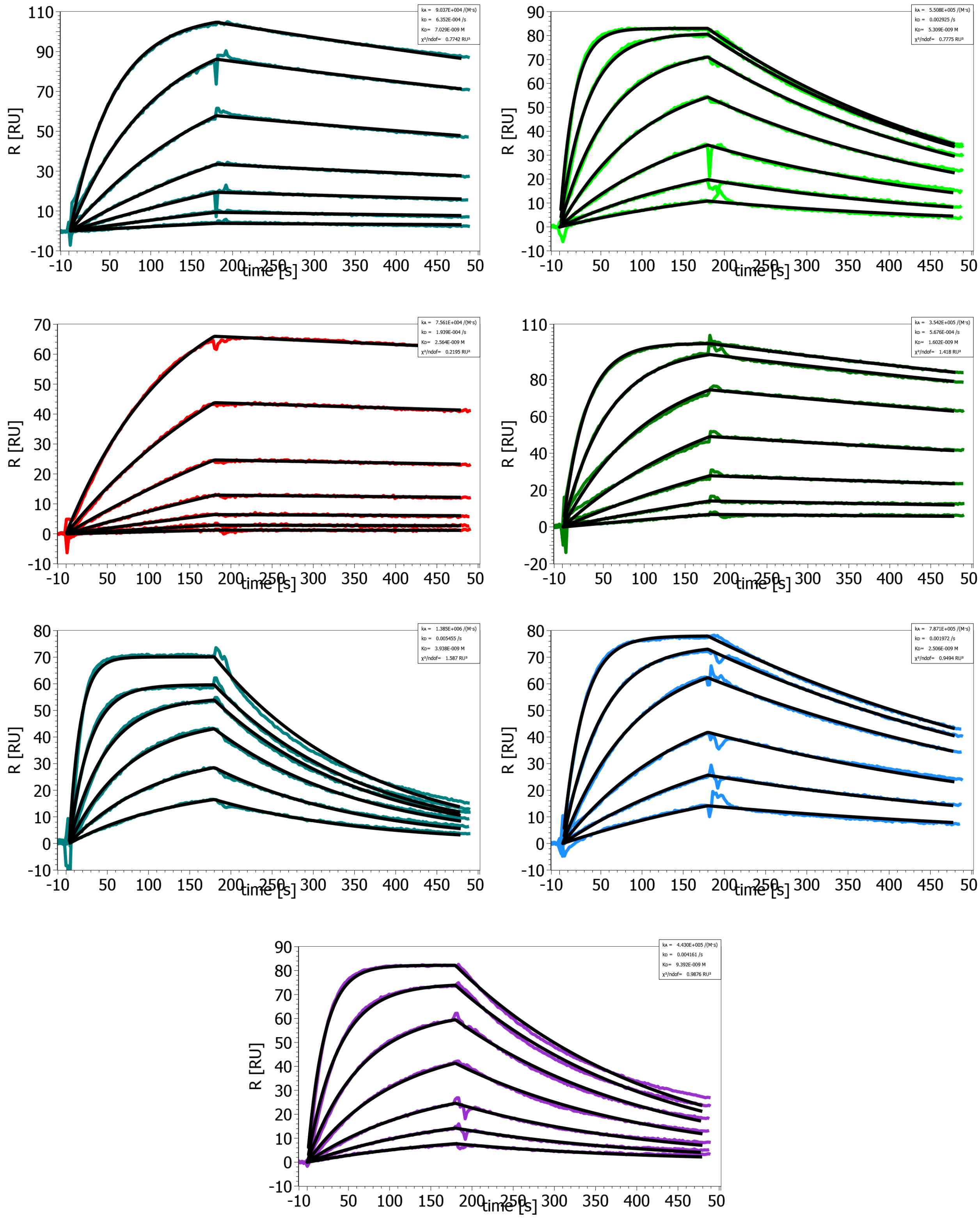

Figure S6: Raw SPR sensograms (colored) and fitted curves (black) for HLA-B*7301 refolded with the P2R peptide and different Fabs identified by phage display.

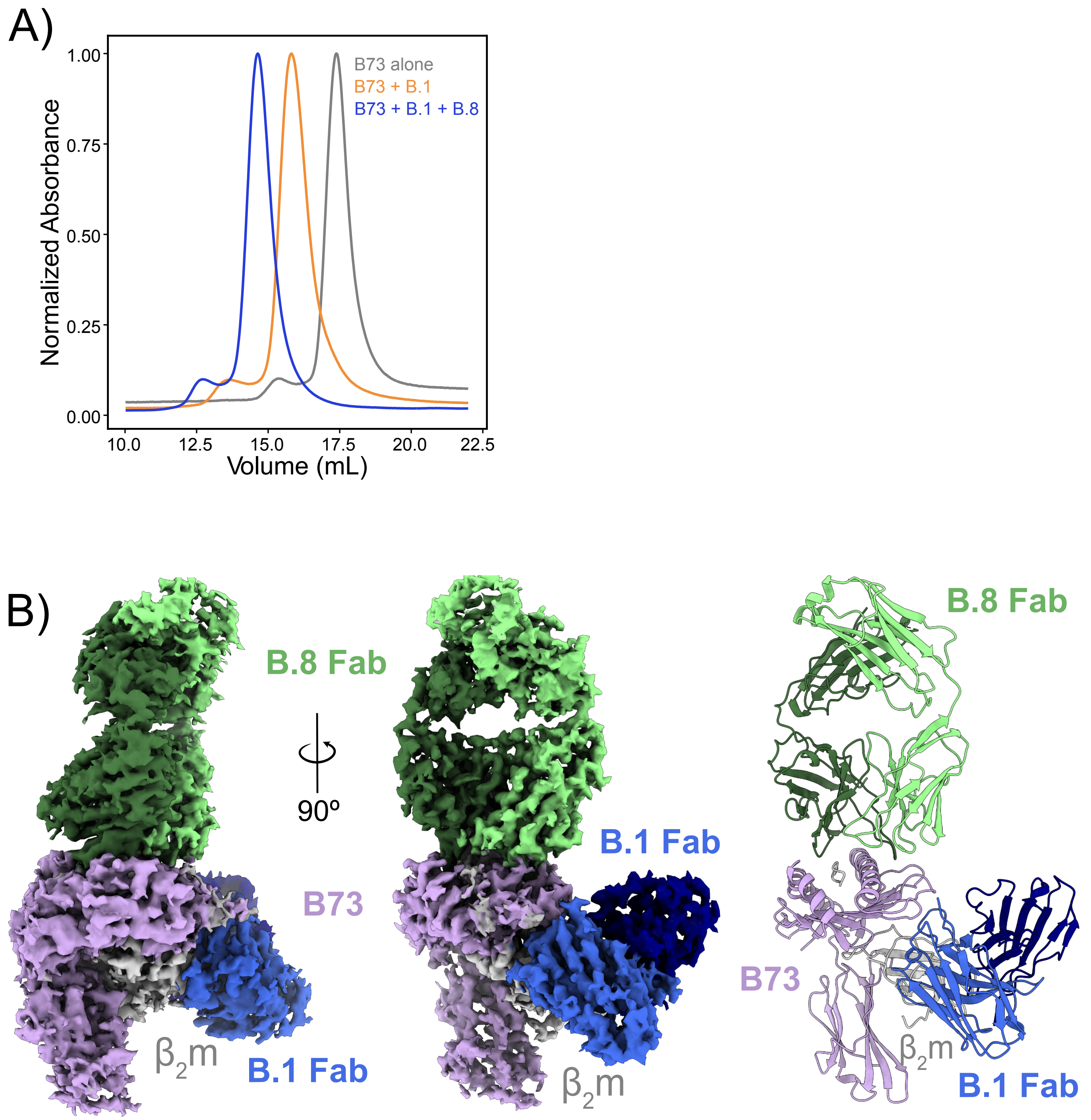

Figure S7: A) Size exclusion chromatography curves showing elution of HLA-B*73:01 following injection of the HLA by itself, with only the B.1 Fab, or with the B.8 and B.1 Fabs. **B)** The masked density map at a threshold of 0.02 of HLA-B*73:01 bound to the P2R peptide and complexed with the B.8 and B.1 Fabs.

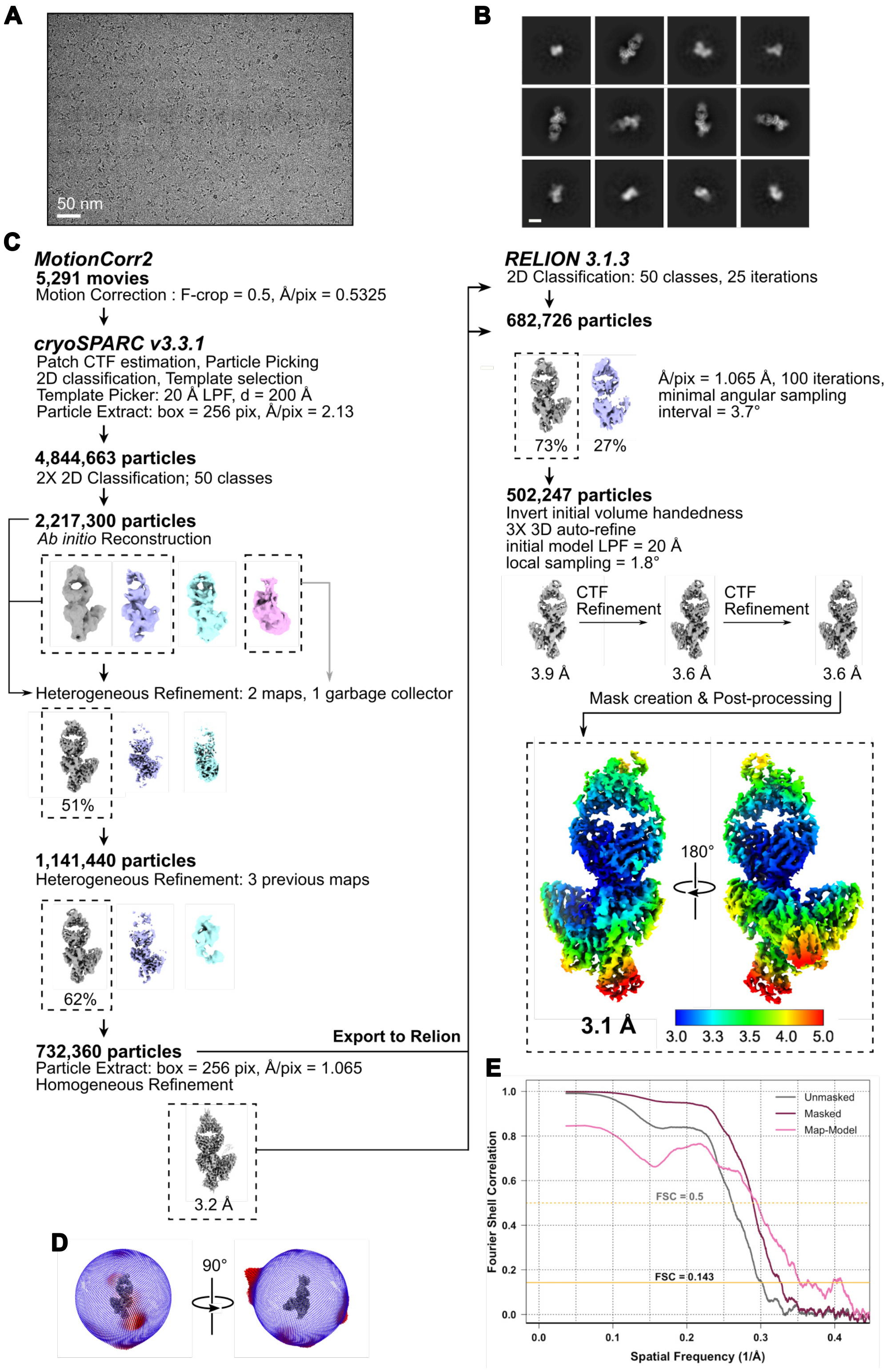

Figure S8: CryoEM processing workflow and intermediate results **A)** Representative cryoEM micrograph from the HLA-B*73:01-B.1-B.8 dataset (n =5,291), collected on a Titan Krios microscope. **B)** A subset of initial, reference-free 2D class averages, following "blob" particle picking. Scale bar is 50 Å. **C)** The image processing workflow for HLA-B*73:01-B.1-B.8. Initial processing was completed using cryoSPARC v3.3.1. Selected particle coordinates were exported to RELION. Exported particles were subjected to two rounds of 2D classification, followed by a round of exhaustive 3D classification in which they were aligned to a 3D volume from cryoSPARC's Homogeneous Refinement. The resulting particles and maps underwent three rounds of CTF refinement and 3D auto-refinement, respectively, followed by solvent masking. The final map is shown with local resolution values calculated in RELION. **D)** Euler angle distributions calculated in RELION are also provided. **E)** Fourier Shell Correlation (FSC) resolutions were calculated in the Phenix suite.

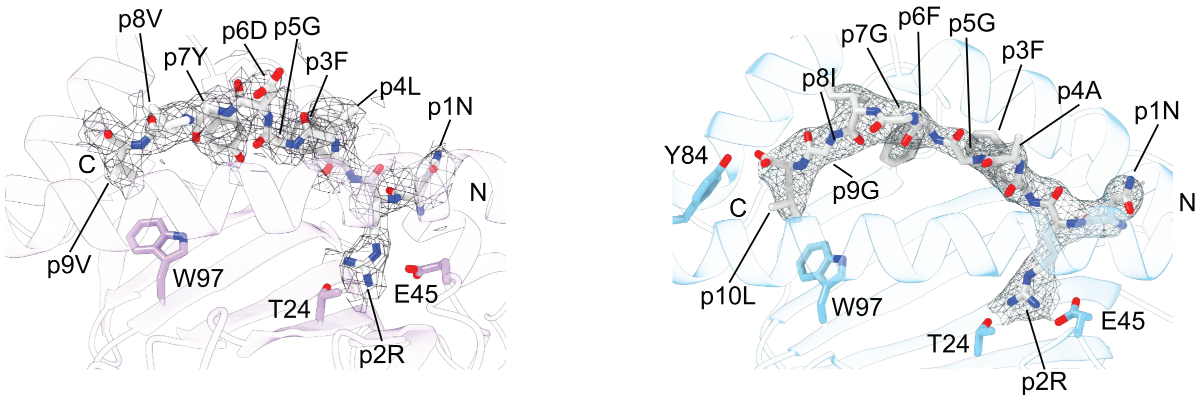

NRFLGDYVV (YVV)

NRFAGFGIGL (KP1)

Figure S9: The mesh surrounding the P2R peptide for the CryoEM (left) structure, representative of the masked map at a threshold of 0.02. The mesh surrounding the KP1 peptide for the X-ray crystal (right) structure, representative of the 2Fo-Fc map restricted to a certain radius surrounding only the peptide itself.

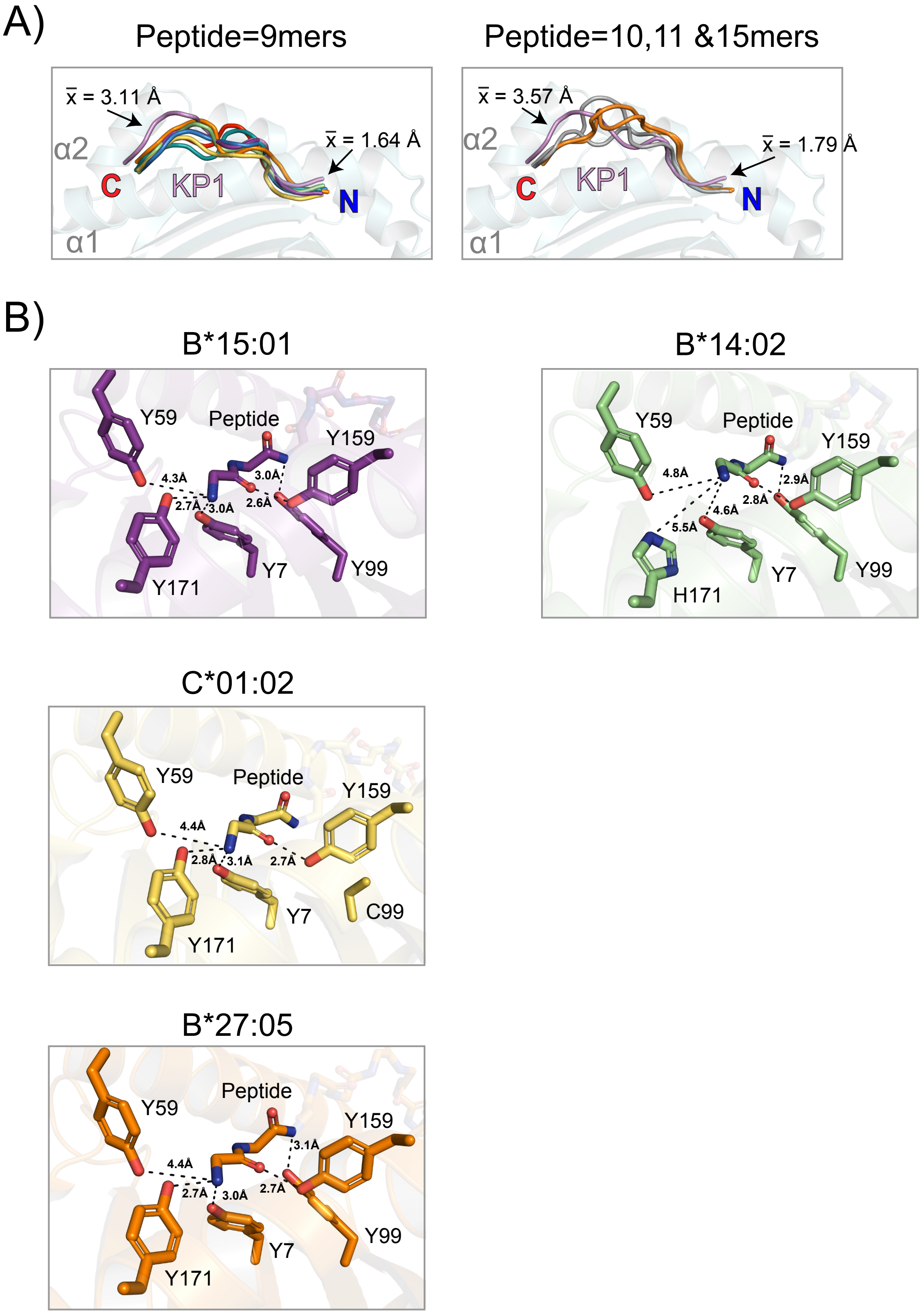

Figure S10: **A)** KP1 bulges out at the C-terminal end of the peptide over the F pocket of B*7301 in a manner not seen in other alleles presenting a variety of 9mers. Colors correspond to allele colors as in Figure 1 and Figure S1. Distances were calculated in PyMol as the distance between alpha carbons at the second to last or first residue within each peptide ligand. Comparing even longer peptides, KP1 still stands out. Distances were calculated as in panel D. **B)** A close up of A pockets are shown for alleles B*1501, B*2705, C*0102, and B*1402. Distances were calculated using PyMol.

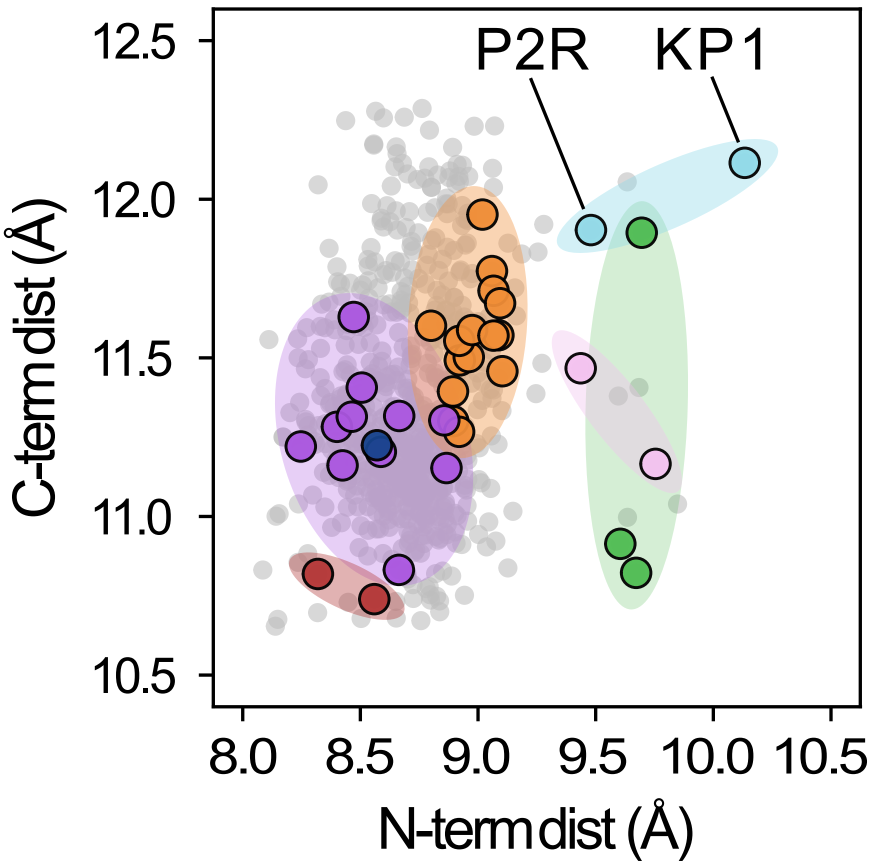

Figure S11: Distances between the Cαs of HLA platform residue 26 and peptide residue 1 (N-term distance) and the HLA platform residue 117 and the peptide omega residue (C-term distance) were calculated for 674 peptide-HLA protein structures curated by the HLA3DB and the two HLA-B*7301 structures from this study. Colored alleles include HLA-B*15:01 (purple), HLA-B*14:02 (green), HLA-B*27:05 (orange), HLA-B*46:01 (red), B*51:01 (pink), HLA-B*73:01 (cyan), and HLA-C*07:02 (navy blue).

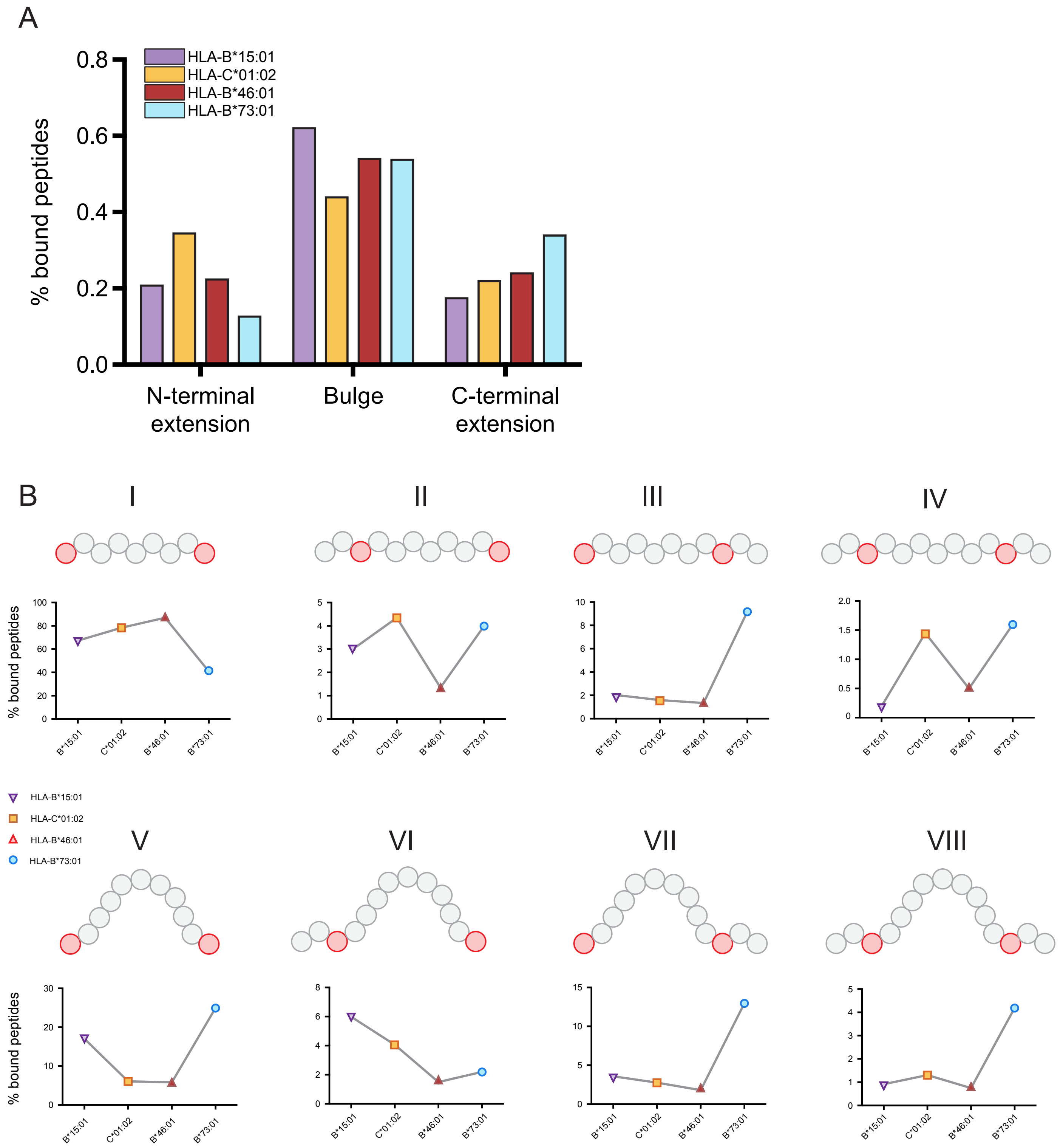

Figure S12: HLA-B*73:01 bound peptides have a connonical HLA class I binding conformation **A)** Histogram showing the percentage of long peptides (11 amino acids or more) from HLA-B*15:01 (purple), HLA-C*01:02 (gold), HLA-B*46:01 (red), and HLA-B*73:01 (cyan) that bind with an N-terminal extended, bulged, or C-terminal extended conformation. **B)** Percentage of all peptides bound by HLA-B*15:01 (purple triange), HLA-C*01:02 (gold square), HLA-B*46:01 (red trangle), and HLA-B*73:01 (cyan circle) adopting the specific conformations (I-VIII) illustrated.

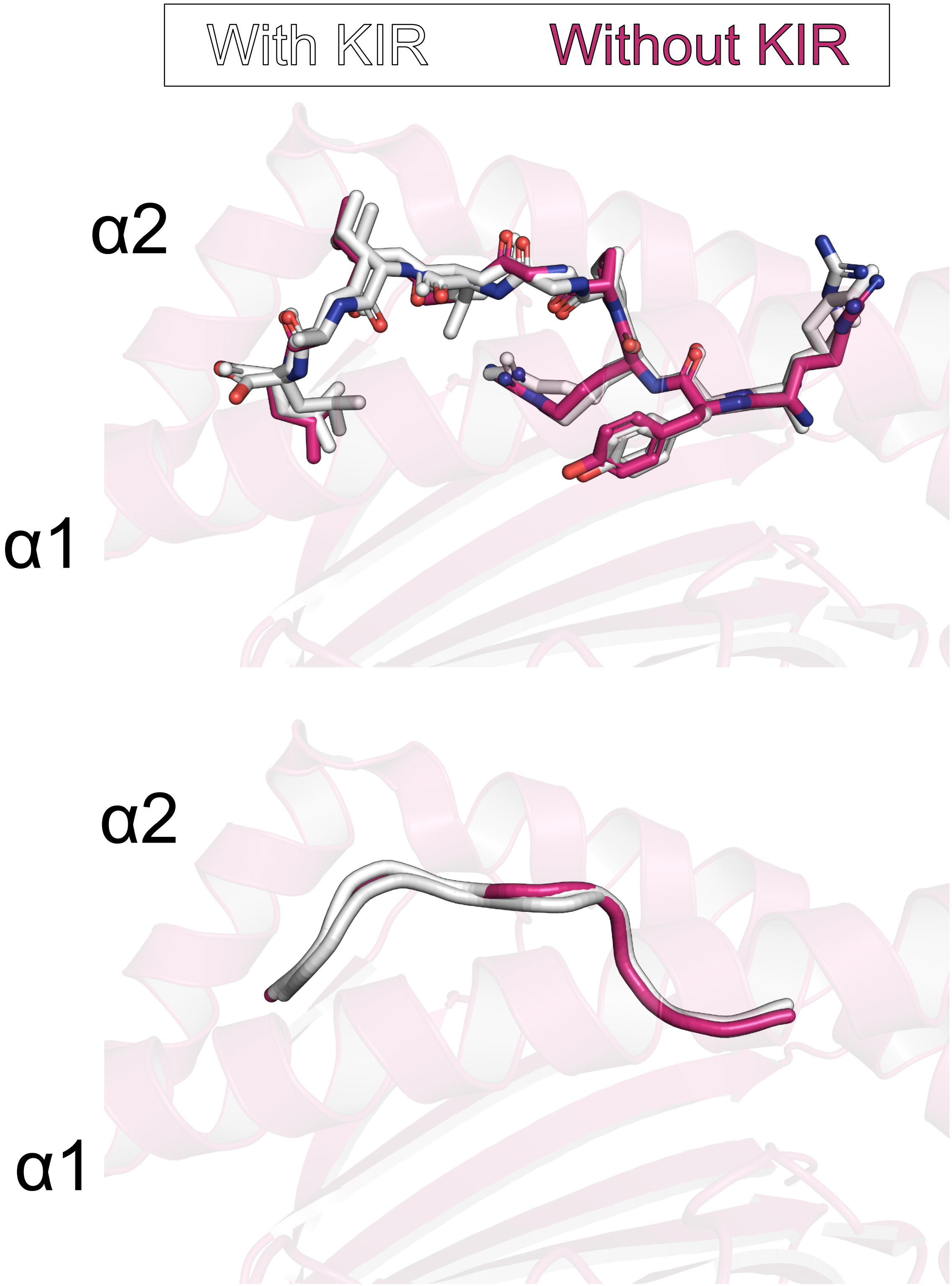

Figure S13: Three structures of C*0702 were aligned using β_2m as a reference. In magenta is a structure of C*0702 presenting RYRPGTVAL without a bound KIR. Two more structures in white are structures of C*0702 presenting the same peptide, bound to KIR2DL2 or KIR2DL3.

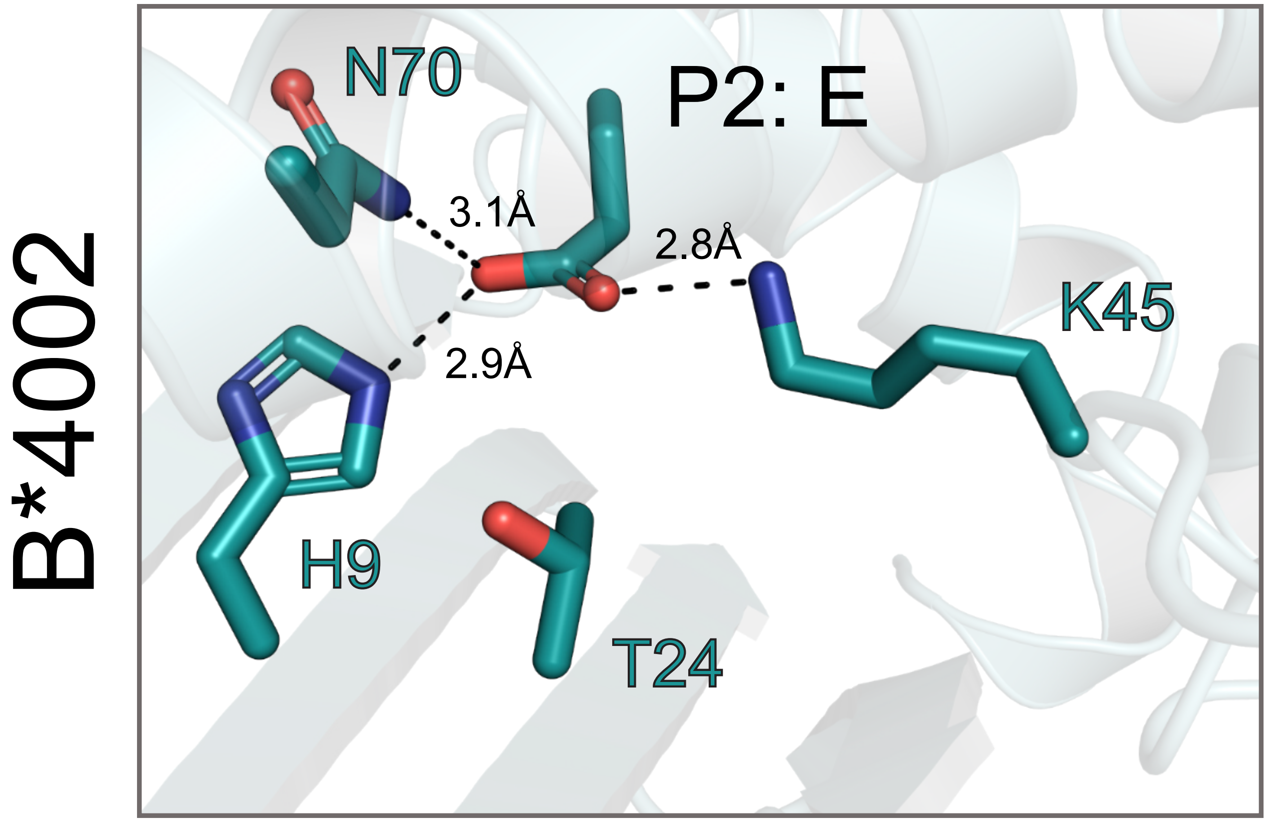

Figure S14: A close up of B pocket of B*4002 show how it uses similar and different residues located on different sides of the binding pocket to accommodate positively or negatively charged anchors.

Table S1. Peptides eludes from B73

Peptide Name	Peptide Sequence (N→C)	Peptide Length
YVV	NRFLGDYVV	9
P2Q	NQFPGFKEV	9
P2E	FEYGGFPPA	9
P2K	PKIVKWDRDM	10
KP1	NRFAGFGIGL	10
KP2	NRFSTPEQA	9
Nona1	NRYHVPVVV	9
Nona2	NRHFGSVVA	9
Deca1	NRYHVPVVVV	10
Deca2	TRLEADEVAA	10
Undeca1	NRYHVPVVVVP	11
Undeca2	DRIMNTFSVVP	11
YAN	YANDFASEY	9
FTM	FTMRLLSPV	9
Dodeca_1	TRFVVDPSIFTA	12
14_1	NRFESHGGGWGYSA	14
Dodeca _2	NRFLEENPDPSA	12
15_1	NRFVGVNASDINYSA	15
Tredec_1	DRYFEADPPGQVA	13
Dodeca_3	NRYYSLWIPEQA	12
Dodeca_4	NRFLEENADPSA	12
Dodeca_5	NRAPEPTPQQVA	12
Tredec_2	NRFSGWYDADLSP	13
Tredec_3	NRFGGSGSQVDSA	13
Tredec_4	NRYGMGTSVERAA	13
Tredec_5	NRFGAQQDTIEVP	13
15_2	NRFSATEVTNKTLAA	15
Tredec_6	HRWSIGKAIDFAA	13
Dodeca_6	DRLKVFDGIPPP	12
Dodeca_7	VRFDSDVGMFVA	12
Tredec_7	NRILWVDENNLTA	13
Dodeca_8	NRFGAQQDTIEV	12
Dodeca_9	NRMSFGEIEEDA	12
Dodeca_10	NRFDGSGPYSTL	12
Dodeca_11	IRFGGKAKEYSP	12

Table S2: Kinetics of B73 specific Fabs.

Fab name	k_{on} (m^{-1} s^{-1})	k_{off} (s^{-1})	k_D (nM)	!^{2} (RU)
B.1	9.0×10^4	6.4×10^{-4}	7.0	0.77
B.2	5.5×10^5	2.9×10^{-3}	5.3	0.77
B.3	7.6×10^4	1.9×10^{-4}	2.6	0.22
B.4	3.5×10^5	5.7×10^{-4}	1.6	1.4
B.8	1.4×10^6	5.4×10^{-3}	3.9	1.6
B.9	7.9×10^5	2.0×10^{-3}	2.0	0.95

Table S3. Data collection, processing and refinement statistics for B.1-B.8-YVV-B2M-B73

**Microscope**	Titan Krios
**Nominal magnification**	81,000
**Voltage (kV)**	300
**Electron exposure (e/Å2)**	60
**Defocus range (μm)**	-2.4 to -1.0
**Pixel size (Å)**	0.5325
**Symmetry imposed**	C 1
**Movies (no.)**	5291
**Software for data collection**	EPU
**Initial "particle" images (no.)**	4,844,663
**Protein particle images (no.)**	2,217,300
**Final particle images (no.)**	502,247
**Map resolution unmasked (Å)**	3.6
**Map resolution masked (Å)**	3.1
**FSC threshold**	0.143
**Refinement**	
Initial model used (PDB code)	8TMU
Model resolution (Å)	3.36
FSC threshold	0.5
**Model composition**	
Non-hydrogen atoms	8073
Protein residues	1040
**B factors ($Å^2$)**	
Protein	86.65
**R.m.s. deviations**	
Bond lengths (Å)	0.003
Bond angles (°)	0.509
**Validation**	
MolProbity score	1.65
Clashscore	5.67
Poor rotamers (%)	0.57
**Ramachandran plot**	
Favored (%)	95.01
Allowed (%)	4.99
Disallowed (%)	0.00
**EMRinger score**	2.82

Table S4. Data processing and refinement statistics for HLA-B*73:01 with KP1 and KIR2DL2

Wavelength	1.0332
Resolution range	54.3 - 2.9 (3.004 - 2.9)
Space group	P 43 21 2
Unit cell	92.69 92.69 200.98 90 90 90
Total reflections	545044 (20136)
Unique reflections	20180 (1973)
Multiplicity	22.4 (16.8)
Completeness (%)	99.97 (100.00)
Mean I/sigma(I)	11.9 (0.9)
Wilson B-factor	68.74
R-merge	0.199 (3.260)
R-meas	0.203 (3.362)
R-pim	0.043 (0.813)
CC1/2	1.00 (0.411)
Reflections used in refinement	20177 (1973)
Reflections used for R-free	999 (94)
R-work	0.2225 (0.3146)
R-free	0.2551 (0.3436)
Number of non-hydrogen atoms	4764
Macromolecules	4645
Ligands	59
Solvent	60
Protein residues	588
RMS(bonds)	0.003
RMS(angles)	0.6
Ramachandran favored (%)	95.33
Ramachandran allowed (%)	4.67
Ramachandran outliers (%)	0
Rotamer outliers (%)	3.08
Clashscore	7.7
Average B-factor	70.86
Macromolecules	70.83
Ligands	80.78
Solvent	63.27
Number of TLS groups	15

Table S5. Peptide:B*7301 contact table

	YVV peptide	Distance (Å)	B*7301	Type	
N1	ASN 1: N	3.31	TYR 7: OH	H-BOND	
	ASN 1		TYR 7	VDW	
	ASN 1: N	3.26	TYR 59: OH	H-BOND	
	ASN 1		TYR 59	VDW	
	ASN 1: OD1	2.42	ARG 62: NH1	H-BOND	
	ASN 1		ARG 62	VDW	
	ASN 1		ASN 63	VDW	
	ASN 1		ILE 66	VDW	*
	ASN 1: O	3.34	TYR 159: OH	H-BOND	
	ASN 1		TYR 159	VDW	
	ASN 1: ND2	2.6	GLU 163: OE2	H-BOND	
	ASN 1		GLU 163	VDW	
	ASN 1		TRP 167	VDW	
	ASN 1		HIS 171	VDW	*
R2	ARG 2		TYR 7	VDW	
	ARG 2		PHE 8	VDW	
	ARG 2		HIS 9	VDW	
	ARG 2: NH1	2.8	THR 24: OG1	H-BOND	
	ARG 2		THR 24	VDW	
	ARG 2		VAL 25	VDW	
	ARG 2		GLY 26	VDW	*
	ARG 2		VAL 34	VDW	*
	ARG 2		ARG 35	VDW	*
	ARG 2		PHE 36	VDW	*
	ARG 2: NE	3.29	GLU 45: OE1	H-BOND	
	ARG 2: NE	3.26	GLU 45: OE2	H-BOND	
	ARG 2: NH2	3.55	GLU 45: OE1	H-BOND	
	ARG 2: NE	3.29	GLU 45: OE1	SALT BR	
	ARG 2: NE	3.26	GLU 45: OE2	SALT BR	
	ARG 2: NH2	3.55	GLU 45: OE1	SALT BR	
	ARG 2: NH2	3.78	GLU 45: OE2	SALT BR	
	ARG 2		GLU 45	VDW	
	ARG 2		ARG 62	VDW	*
	ARG 2		ASN 63	VDW	
	ARG 2		ILE 66	VDW	
	ARG 2		CYS 67	VDW	
	ARG 2		TYR 99	VDW	
F3	PHE 3		ILE 66	VDW	
	PHE 3: N	2.84	TYR 99: OH	H-BOND	
	PHE 3: O	3.41	TYR 99: OH	H-BOND	
	PHE 3		TYR 99	VDW	
	PHE 3		GLN 155	VDW	
	PHE 3		LEU 156	VDW	
	PHE 3		TYR 159	VDW	
L4	LEU 4		ILE 66	VDW	
	LEU 4		ALA 69	VDW	
G5	GLY 5		ILE 66	VDW	
	GLY 5		LYS 70	VDW	
	GLY 5		THR 73	VDW	
	GLY 5		LEU 156	VDW	
D6	ASP 6		THR 73	VDW	
Y7	TYR 7: OH	3.12	ASN 114: ND2	H-BOND	
	TYR 7		THR 73	VDW	
	TYR 7		ASN 114	VDW	
	TYR 7		PHE 116	VDW	
	TYR 7		TRP 147	VDW	
	TYR 7		VAL 152	VDW	
	TYR 7		LEU 156	VDW	
V8	VAL 8		THR 73	VDW	
	VAL 8		VAL 76	VDW	
	VAL 8		ASN 80	VDW	
	VAL 8: O	3.29	LYS 146: NZ	H-BOND	
	VAL 8		LYS 146	VDW	
	VAL 8: O	3.01	TRP 147: NE1	H-BOND	
	VAL 8		TRP 147	VDW	
V9	VAL 9		VAL 76	VDW	*
	VAL 9		GLY 77	VDW	
	VAL 9		ASN 80	VDW	
	VAL 9		LEU 81	VDW	
	VAL 9		TRP 95	VDW	
	VAL 9		TYR 123	VDW	*
	VAL 9		THR 143	VDW	
	VAL 9		LYS 146	VDW	
	VAL 9		TRP 147	VDW	

	KP1 peptide	Distance (Å)	B*7301	Type	
N1	ASN 1		TYR 7	VDW	
	ASN 1		TYR 59	VDW	
	ASN 1		ARG 62	VDW	
	ASN 1: OD1	3.48	ARG 62: NE	H-BOND	
	ASN 1		ASN 63	VDW	
	ASN 1: OD1	2.76	ASN 63: ND2	H-BOND	
	ASN 1: O	2.37	TYR 159: OH	H-BOND	
	ASN 1		TYR 159	VDW	
	ASN 1: ND2	3.33	GLU 163: OE2	H-BOND	
	ASN 1		GLU 163	VDW	
	ASN 1		TRP 167	VDW	
R2	ARG 2		TYR 7	VDW	
	ARG 2		PHE 8	VDW	
	ARG 2		HIS 9	VDW	
	ARG 2: NH2	2.2	THR 24: OG1	H-BOND	
	ARG 2		THR 24	VDW	
	ARG 2		VAL 25	VDW	
	ARG 2: NH1	2.57	GLU 45: OE1	H-BOND	
	ARG 2: NH1	2.57	GLU 45: OE1	SALT BR	
	ARG 2: NH1	2.97	GLU 45: OE2	SALT BR	
	ARG 2		GLU 45	VDW	
	ARG 2: N	2.78	ASN 63: OD1	H-BOND	
	ARG 2		ASN 63	VDW	
	ARG 2		ILE 66	VDW	
	ARG 2		CYS 67	VDW	
	ARG 2		TYR 99	VDW	
	ARG 2		TYR 159	VDW	*
F3	PHE 3: N	2.99	TYR 99: OH	H-BOND	
	PHE 3		ILE 66	VDW	
	PHE 3		TYR 99	VDW	
	PHE 3		GLN 155	VDW	
	PHE 3		LEU 156	VDW	
	PHE 3		TYR 159	VDW	
A4	ALA 4		ILE 66	VDW	
F6	PHE 6		PHE 116	VDW	
	PHE 6		TRP 147	VDW	
	PHE 6		VAL 152	VDW	
	PHE 6		GLN 155	VDW	
	PHE 6		LEU 156	VDW	
G7	GLY 7		GLN 71	VDW	
I8	ILE 8		GLN 71	VDW	
	ILE 8		TYR 105	VDW	
	ILE 8		LYS 146	VDW	
	ILE 8		TRP 147	VDW	
	ILE 8		ALA 150	VDW	
	ILE 8		VAL 152	VDW	
G9	GLY 9		GLN 71	VDW	
	GLY 9: O	3.32	TRP 147: NE1	H-BOND	
	GLY 9		TRP 147	VDW	
L10	LEU 10		GLY 77	VDW	
	LEU 10: O	3.69	ASN 80: ND2	H-BOND	
	LEU 10		ASN 80	VDW	
	LEU 10		LEU 81	VDW	
	LEU 10: O	2.77	TYR 84: OH	H-BOND	*
	LEU 10: OXT	2.53	TYR 84: OH	H-BOND	*
	LEU 10		TYR 84	VDW	*
	LEU 10		TRP 95	VDW	
	LEU 10		THR 143	VDW	
	LEU 10: OXT	3.7	LYS 146: NZ	H-BOND	
	LEU 10: OXT	3.7	LYS 146: NZ	SALT BR	
	LEU 10		LYS 146	VDW	
	LEU 10		TRP 147	VDW	
